# Functional and genetic diversity of native rhizobial isolates nodulating cowpea (*Vigna unguiculata* L. Walp.) in Mozambican soils

**DOI:** 10.1038/s41598-021-91889-7

**Published:** 2021-06-17

**Authors:** Margarida G. Simbine, Mustapha Mohammed, Sanjay K. Jaiswal, Felix D. Dakora

**Affiliations:** 1 Department of Crop Sciences, Tshwane University of Technology, Private Bag X680, Pretoria, 0001 South Africa; 2 Department of Crop Science, University for Development Studies, P.O. Box TL1882, Tamale, Ghana; 3 Department of Chemistry, Tshwane University of Technology, Arcadia campus, 175 Nelson Mandela Drive, Private Bag X680, Pretoria, 0001 South Africa

**Keywords:** Symbiosis, Soil microbiology

## Abstract

Identification and symbiotic characterization of indigenous rhizobial isolates are the basis for inoculant formulations needed for sustainable grain legume production. This study screened for morpho-genetic diversity of indigenous cowpea nodulating rhizobia in farmers’ fields across two contrasting agroecological zones of Northern Mozambique. The photosynthetic function induced by the isolates in their homologous cowpea was assessed. The results showed high genetic variability among the isolates based on morphology and ERIC-PCR fingerprinting. The trap cowpea genotype did not influence the diversity of isolates collected from the two different agroecologies, suggesting that the cowpea-rhizobia compatibility may be conserved at species level. Phylogenetic analysis of the 16S rRNA gene assigned representative rhizobial isolates to species in the *Bradyrhizobium* and *Rhizobium* genera, with some isolates showing high divergence from the known reference type strains. The isolates from both agroecologies highly varied in the number and biomass of nodules induced in the homologous cowpea, resulting in variable plant growth and photosynthetic activities. A total of 72% and 83% of the isolates collected from the agroecological zones 7 and 8 were respectively classified as highly effective candidates with > 80% relative effectiveness compared to plants fertilized with nitrate, indicating that elite native strains populated the studied soils. Moreover, the top 25% of high N_2_-fixing isolates from the two agroecologies recorded relative effectiveness ranging from 115 to 154%, values higher than the effectiveness induced by the commercial *Bradyrhizobium* sp. strain CB756. These strains are considered as having potential for use in inoculant formulations. However, future studies should be done to assess the ecologically adaptive traits and symbiotic performance under field conditions.

## Introduction

Nitrogen is one of the essential macro nutrient elements required for plant growth, synthesis of macromolecules such as chlorophyll needed for photosynthesis^1,2^ and other biomolecules such as Rubisco which reduces CO_2_ during photosynthesis^3^. Inadequate level of mineral N in soil can limit plant growth and reproduction through inhibition of chlorophyll and Rubisco formation with reduced carbohydrate production^4,5^. With adequate mineral N, however, plant growth can easily be developed with sufficient sunlight, water, carbon dioxide and nutrients via photosynthesis^6–8^.

Soil depletion of nitrogen and phosphorus is the major nutritional stress causing a decline in the per capita food production in Sub-Saharan Africa and other parts of the world^9,10^. The annual loss of N from soils in Mozambique is estimated at > 60 kg ha^−1^^11^. As a result, there is a need to replenish soil N through application of mineral fertilizers. However, the high cost of N fertilizers in Africa makes them inaccessible to resource-poor smallholder farmers^12–14^. Fortunately, inclusion of legumes in cropping systems has been documented as a cheaper partial alternative to N fertilizers due to their ability to fix N_2_ when in symbiosis with rhizobia^15,16^. Nitrogen fixation by bacteroids in root nodules ensures the supply of high amounts of N required for meeting plant growth^17,18^ and have turnover effects on companion or following non-leguminous crops when grown simultaneously or in rotation^15,19,20^. Thus, food legumes are considered a necessary component of cropping systems in low-input agriculture at nearly no cost to farmers^21,22^.

The cowpea-*Rhizobium* symbiosis is highly efficient as it contributes more than 60% of the legume’s N requirement, and up to 200 kg N ha^−1^ to the cropping system^21,23–25^. However, the efficiency of N_2_ fixation differ for various legume/rhizobia combinations and for soil and climatic conditions^26–29^. For example, indigenous rhizobia may be abundant in soils and infected root, but yet be inefficient or exhibit low N_2_-fixing efficiency, leading to retarded plant growth^30^. Under such conditions, inoculation of the legume with highly effective rhizobial strains can improve N_2_ fixation, plant growth and grain yield^31,32^. However, formulation of effective rhizobial inoculants requires screening and selection of highly effective and competitive native N_2_-fixing isolates compatible with the host plant and better suited for N_2_ fixation in a wide range of environments^28^. The ability of rhizobial isolates to form efficient symbiosis with recommended host legume has been considered a major criterion used for selection of rhizobial strains for inocula production^33,34^.

In Mozambique, there is a rising interest in the use of rhizobial inoculants for enhanced legume production^29,32,35–37^. However, the inoculants used there are imported mainly from South Africa and Kenya, and are formulated using exotic rhizobial strains. Although recent studies have demonstrated the presence of cowpea-compatible rhizobia in Mozambican soils^35^, there is limited information regarding their N_2_-fixing efficiency. Earlier reports have revealed the presence of efficient N_2_-fixing rhizobia nodulating soybean in Mozambique^36,37^. This suggests the need of screening for efficient symbionts for other grain legumes, including cowpea. Furthermore, despite the presence of highly diverse cowpea-nodulating rhizobia in Africa and elsewhere^38–44^, little is known about their diversity in the Mozambican legume production fields.

The aim of this study was to assess morpho-genetic diversity of cowpea-nodulating rhizobia in Mozambican soils, and ordering them according to their symbiotic effectiveness. The study also includes a comparison between N fertilization and a commercial inoculant of *Bradyrhizobium* sp. strain CB756 currently under use in this area and further assessment of variations in photosynthetic functioning induced by inoculation with diverse native rhizobial isolates on cowpea, their homologous host.

## Materials and methods

### Work locations

**Figure 1:**
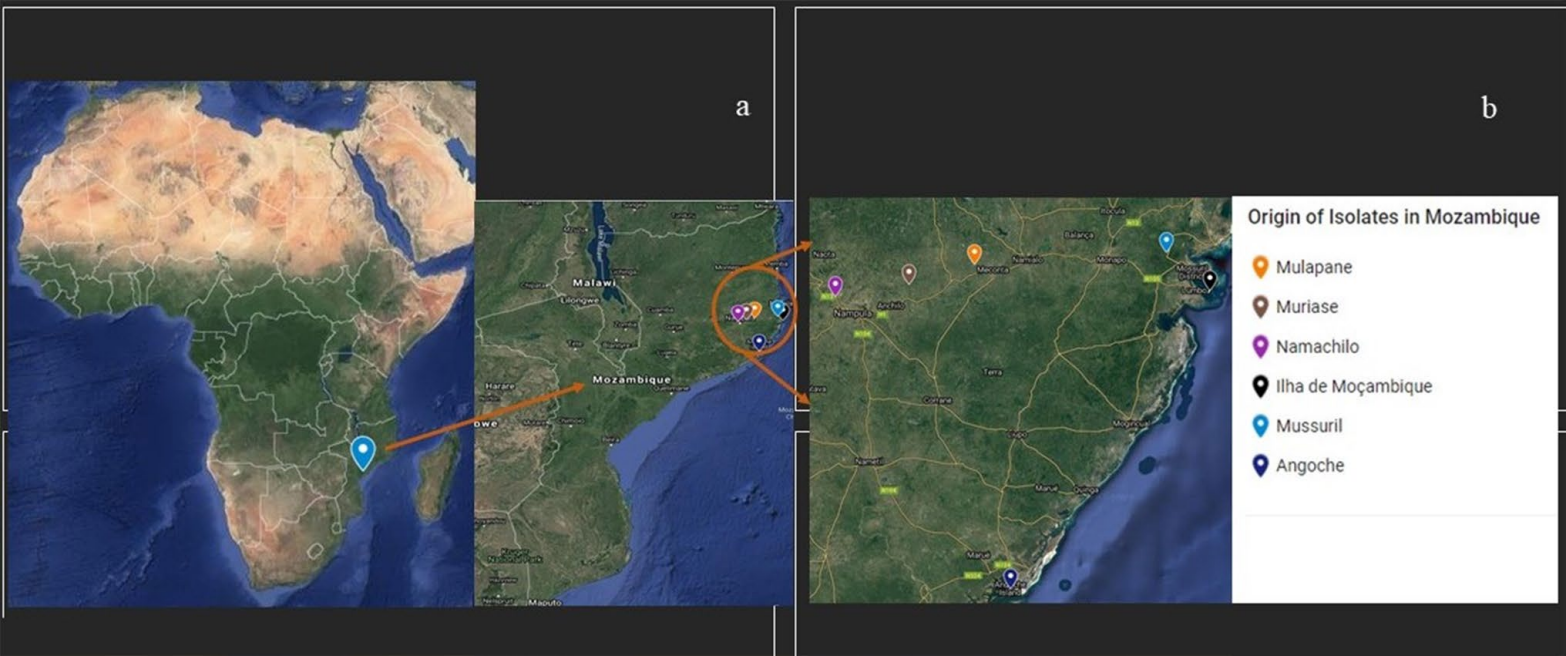
A map showing (**a**) the geographic origins of the cowpea isolates from Mozambique that were used in this study. The sampling locations are (**b**) magnified and indicated by means of coloured markers/pointers that are defined using legends. The map was created using Google Maps (https://www.google.com/maps) by adding markers corresponding to the GPS coordinates of the sampling locations. The resulting map was cropped to obtain the areas consisting of the sampling locations used in this study. The detailed map is available at https://www.google.com.gh/maps/@-14.5660144,38.6943858,6.5z/data=!4m2!6m1!1s15WALAjIoz1lm-E2-u4QD4MatOlcDYdMD?hl=en (last accessed on 8th January, 2021).

The bacterial strains used in this study were isolated from root nodules of cowpea collected from farmers’ fields in Mulapane (Latitude: − 14.93521266 and Longitude: 39.7655745), Meconta district, Muriase (Latitude: − 15.01492147 and Longitude: 39.49549732) and Namachilo (Latitude: − 15.062666 and Longitude: 39.193373) in the Rapale district, all three study sites in the Agroecological zone 7 (AEZ 7), along with others from Ilha de Moçambique (Latitude: − 15.036449 and Longitude: 40.732331), Mussuril (Latitude: − 14.886520 and Longitude: 40.553400) and Angoche (Latitude: − 16.216266 and Longitude: 39.914466) districts, in the Agroecological zone 8 (AEZ 8) in the Nampula Province, Northern Mozambique (Fig. 1).

The cowpea genotypes IT-16, IT-18, Sudan-1, T-1263 and T99K-529-1 along with the locally cultivated landrace Namuruwa were used as trap hosts across the locations in AEZ 7. However, only the local landrace Namuruwa was planted as a trap host in AEZ 8.

The two agroecologies were chosen for their contrast in the amounts of rainfall. The agroecological zone 7 (AEZ 7) is characterized by an annual rainfall of 1000 to 1400 mm, with an evapotranspiration ranging from 100 to 1400 mm and annual daytime temperatures of 24 to 26 °C. The predominant soils in this AEZ 7 are ultisol and oxisol. In contrast, AEZ 8 receives an annual rainfall of 800 to 1200 mm, experiences much higher evapotranspiration (> 1400 mm) and average daytime temperatures higher than 25 °C. The predominant soil type in AEZ 8 is alfisol.

### Sampling of root nodules and bacterial isolation

Root nodules of cowpea used in this study were sampled from 27 farmers’ fields, 12 fields from AEZ 7 and 15 from AEZ 8. All the 27 fields were of unknown history of rhizobial inoculation. The nodules were collected between flowering and early podding stage. Five plants were dug up from each farmer’s field using a hand hoe, the nodulated roots were placed in plastic bags, and transported to the laboratory in a cooler box containing ice. The roots were washed, nodules detached and stored on silica gel covered by a layer of sterile cotton wool contained in plastic vials at 4 °C prior to bacterial isolation, as described by Somasegaran and Hoben^45^.

The bacterial isolates got named to be distinguished as isolated by the academic institution of the study with a prefix (TUT = Tshwane University of Technology) followed by the host plant species (Vu = *Vigna unguiculate* L. Walp.) from which the nodules were obtained, the geographic origin (location) of the nodule (AG = Angoche, IM = Ilha de Moçambique, MS = Mussuril, ML = Mulapane, MU = Muriase, NM = Namachilo), and then an assigned serial number.

### Authentication of isolates in glasshouse

The isolates were tested for their ability to form root nodules on the homologous cowpea host (genotype IT-18) in a naturally lit glasshouse (with plants depending on naturally prevailing sunlight) at the Tshwane University of Technology, South Africa. The isolates from AEZ 7 were evaluated in two separate experiments: 1 and 2 in 2015, while the isolates from AEZ 8 were tested in one experiment in 2016. Surface-sterilised seeds^45^ of cowpea (genotype IT-18) were planted in autoclaved sand contained in sterile plastic pots. Two seeds were planted per pot and thinned to one after germination. The seedling in each pot was aseptically inoculated in the laminar flow hood with 1.0 ml of rhizobial culture (10^9^ cells ml^−1^) grown in yeast extract mannitol broth to the exponential phase. Three replicate pots were used for each isolate, arranged in randomised complete blocks. The seedlings were raised in the glasshouse at an average day and night temperature of 28 °C and 20 °C, respectively. The plants were supplemented with equal amounts of N-free nutrient solution^46^ and sterile distilled water when necessary. Uninoculated plants were included as negative control, while plants inoculated with the commercial *Bradyrhizobium* sp. strain CB756 or supplied with 5 mM KNO_3_ were included as positive controls. At 60 days after sowing, the plants were uprooted and assessed for nodulation. Plants with dark green leaves and internal reddish/pink nodule colouration indicated effective nodulation and most likely efficient symbiotic N fixation.

### Symbiotic effectiveness of the rhizobial isolates

To assess the symbiotic effectiveness of the rhizobial isolates, photosynthetic rates (A) and stomatal conductance (gs) were measured on young and fully expanded trifoliate leaves of 60-day-old cowpea plants grown in the glasshouse. Gas exchange measurements were measured using a portable photosynthesis system (LI 6400XT, version 6.2, Lincoln, Nebraska, USA) as described in Mohammed et al.^44^. The plants were harvested and assessed for nodule number and dry weight and shoot biomass. The nodules and shoots were separately oven-dried at 60 °C for 48 h and weighed. The use of plant parts in the present study complied with international, national, and/or institutional guidelines^47^. The relative effectiveness of each rhizobial isolate with the N-fertilized counterparts was calculated as^37^:

$$ {\text{RE}} = \frac{{{\text{Shoot}}\;{\text{dry}}\;{\text{matter}}\;{\text{of}}\;{\text{inoculated}}\;{\text{plant}}}}{{{\text{Shoot}}\;{\text{dry}}\;{\text{matter}}\;{\text{of}}\;{\text{N - fertilized}}\;{\text{plant}}}} \times 100 $$

The isolates were categorised as highly effective (> 80% RE), moderately effective (50 to 80% RE), lowly effective (35 to 49% RE) and ineffective (< 35% RE).

### Morphological characteristics of rhizobial isolates

To assess colony morphology, the authenticated rhizobial isolates were re-streaked on yeast mannitol agar (YMA) plates, incubated at 28 °C and monitored for bacterial growth from 2 to 12 days. The morphological characteristics recorded included colony colour (white, milky, watery or translucent), texture (gummy, elastic or dry), shape (flat, circular, domed or irregular) and size (colony diameter). The colonies which took < 5 days to appear on YMA plates were classified as fast growers as opposed to the slow growers which took ≥ 5 days to appear^45,48^.

### Bacterial genomic DNA extraction and ERIC-PCR fingerprinting

The genomic DNA of each rhizobial isolate grown in YMB (1 × 10^9^ rhizobia cells ml^−1^) was extracted using a GenElute Bacterial Genomic DNA kit according to the manufacturer’s instructions (Sigma Aldrich, USA), then subjected to ERIC-PCR in a Thermal cycler (T100 Bio-Rad, USA) in a 25 ul reaction volume as described by Ibny et al.^49^. The ERIC-PCR products were electrophoresed in 1.2% agarose gel stained with ethidium bromide (1 µg ml^–1^) in 1X TAE buffer. A standard molecular marker (GeneDirex 1 kb ladder) was included to estimate the weight of bands. The gels were photographed under UV trans-illuminator using the gel documentation system Gel Doc XR +, Bio-Rad, USA.

Cluster analysis based on the ERIC-PCR banding patterns was done with the UPGMA (Unweighted Pair Group Method with Arithmetic mean) algorithm to generate a dendrogram using the software Bionumerics (version 8.0).

### PCR amplification of the 16S rRNA and sequencing

To identify the rhizobial symbionts of cowpea in this study, representative isolates were subjected to phylogenetic analysis based on the 16S rRNA gene. For this, the selected isolates were subjected to PCR amplification of the 16S rRNA gene using primer pairs in a 25 µl reaction mixture as described previousely^49^. To confirm the PCR products, gel electrophoresis was carried out in 1% agarose gel stained with ethidium bromide in TAE buffer at 85 V for 1 h.

The amplified PCR products were purified using PCR Clean-up kit (NEB, USA) by following the manufacturer’s instructions. The purified amplified products were sent to Macrogen (Netherlands) for sequencing of one strand of the 16S rRNA gene. Thereafter, the quality of sequences was checked using the software BioEdit 7.0.9.0^50^. The BLASTn program was used to search for closely related species in the NCBI database^51^. Pairwise and multiple sequence alignments, and phylogenetic trees were constructed using trimmed sequences of uniform lengths by means of the maximum likelihood statistical method using MEGA 7 software^52^. The robustness of branching was estimated using 1000 bootstrap replicates^53^. The sequences were deposited in the NCBI to obtain accession numbers (MZ007813–MZ007845).

### Statistical analysis

The quantitative data on symbiotic effectiveness and gas-exchange measurements were subjected to analysis of variance using GenStat, 11th Edition. The datasets were normally distributed with mean ≈ median, kurtosis and skewness values respectively ranging from − 0.80 to + 1.33 and − 0.58 to + 1.16. Where significant differences were expressed, the means were compared using the Duncan’s multiple range test at p ≤ 0.05. Pearson’s correlation analysis was performed to determine the relationships between variables. The variability in the mean values of the symbiotic and gas-exchange parameters was explored using Box and whisker plots.

## Results

### Morphological characterisation of the rhizobial isolates

A total of 203 isolates were obtained from root nodules of cowpea collected from various locations in two agroecological zones in Mozambique (Fig. 1). Of them, 175 isolates (99 from AEZ 7 and 76 from AEZ 8) induced root nodulation. Hundred and thirty-seven isolates (79 from AEZ 7 and 58 from AEZ 8) were used for evaluation of the symbiotic effectiveness and gas-exchange capacities.

Table 1 shows that the isolates differed in their cultural characteristics. At each agroecological district, 90% of the isolates were found to be slow-growers and took ≥ 5 days to appear on YMA plates, while 10% exhibited fast growth. Furthermore, 62% of the isolates from AEZ 7 and 76% from those of AEZ 8 had smaller colony size of diameter ≤ 1.5 mm, while 38% from AEZ 7 and 24% from AEZ 8 induced colony diameters between 1.5 and 4.5 mm. Most colonies, 45 from AEZ 7 and 43 from AEZ 8, were circular in shape. The other isolates were flat, domed or irregular. Besides, most isolates (71% from AEZ 7 and 78% from AEZ 8) had milky colonies, while the remaining isolates were translucent, whitish, watery or yellowish in colour. Moreover, 95% of isolates from AEZ 7 and 93% from AEZ 8 were gummy in texture while only 5% and 7%, respectively, exhibited dry colony texture.

**Table 1:** Summary of the morpho-physiological characteristics of native rhizobial strains tested.

Characteristic	Number and proportion of isolates from	
	AEZ 7	AEZ 8
**Growth (days)**		
< 5 (fast)	8 (10.13%)	6 (10.34%)
≥ 5 (slow)	71 (89.87%)	52 (89.66%)
**Size (diameter)**		
≤ 1.5 mm	49 (62.03%)	44 (75.86%)
1.5–4.5 mm	30 (37.97%)	14 (24.14%)
**Shape**		
Irregular	22 (27.85%)	12 (20.69%)
Domed	11 (13.92%)	3 (5.17%)
Circular	45 (56.96%)	43 (74.14%)
Flat	1 (1.27%)	–
**Colour**		
Milky	56 (70.89%)	45 (77.59%)
Translucent	5 (6.33%)	2 (3.45%)
Whitish	9 (11.39%)	6 (10.34%)
Watery	6 (7.59%)	4 (6.90%)
Yellowish	3 (3.80%)	1 (1.72%)
**Texture**		
Gummy	75 (94.94%)	54 (93.10%)
Dry	4 (5.06%)	4 (6.90%)

*AEZ* agroecological zone.

### Genetic diversity of the rhizobial isolates

**Figure 2:**
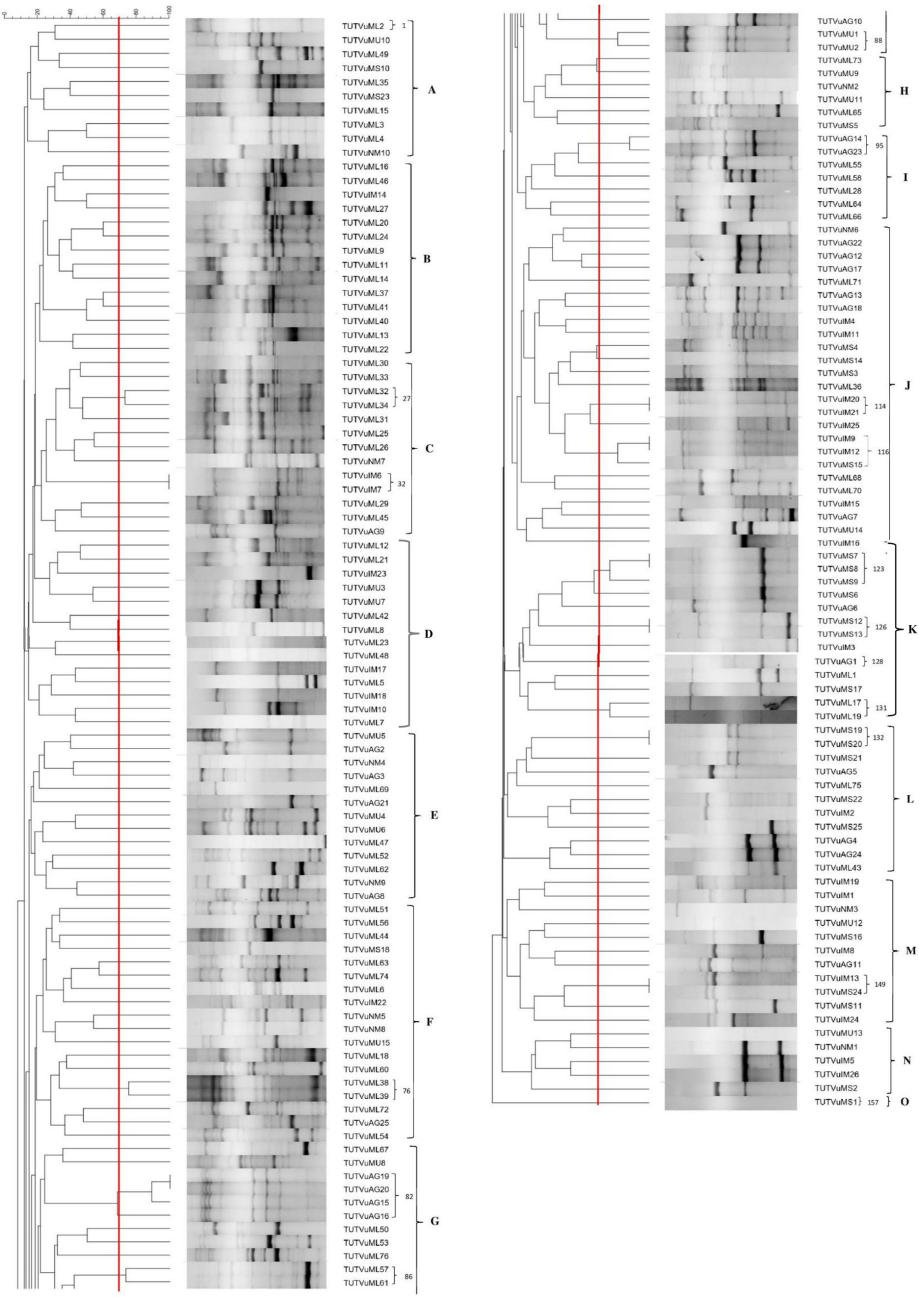
Dendrogram constructed from the ERIC PCR fingerprints of 175 cowpea-nodulating rhizobial isolates from two agroecological zones of Mozambique. Major clusters are labelled with Bold alphabets. The cowpea isolates having distinct ERIC PCR profiles at a cut-off point of 70% similarity (indicated by red line) are labelled using Arabic numerals. Where consecutive isolates possess different ERIC PCR profiles, the numbering is skipped and continued at the next group of isolates.

The ERIC-PCR fingerprinting for the 175 authenticated rhizobial isolates showed variable band patterns. The dendrogram constructed from their banding patterns grouped them into 157 ERIC-PCR types when considered at a 70% similarity cut-off point, which were further grouped into 15 major Clusters (A–O) (Fig. 2). Except for Clusters A and B, which exclusively comprised isolates from AEZ 7, the remaining clusters were heterogeneous, consisting of isolates from the two agroecologies. Interestingly, within the heterogenous major Clusters, the isolates from the same agroecology were largely grouped in proximity, albeit a few exceptions (Fig. 2). Most isolates which were identical based on the ERIC-PCR fingerprints originated from the same location and agroecology, albeit a few exceptions. For example, except for isolates TUTVuIM13 and TTVuMS23 respectively from Ilha de Moçambique and Mussuril in AEZ 8 which were identical in Cluster M, the other identical isolates in Clusters C, G, J, K and L originated from the same location within their respective agroecologies (Fig. 2).

### Phylogeny of isolates based on 16S rRNA gene

**Figure 3:**
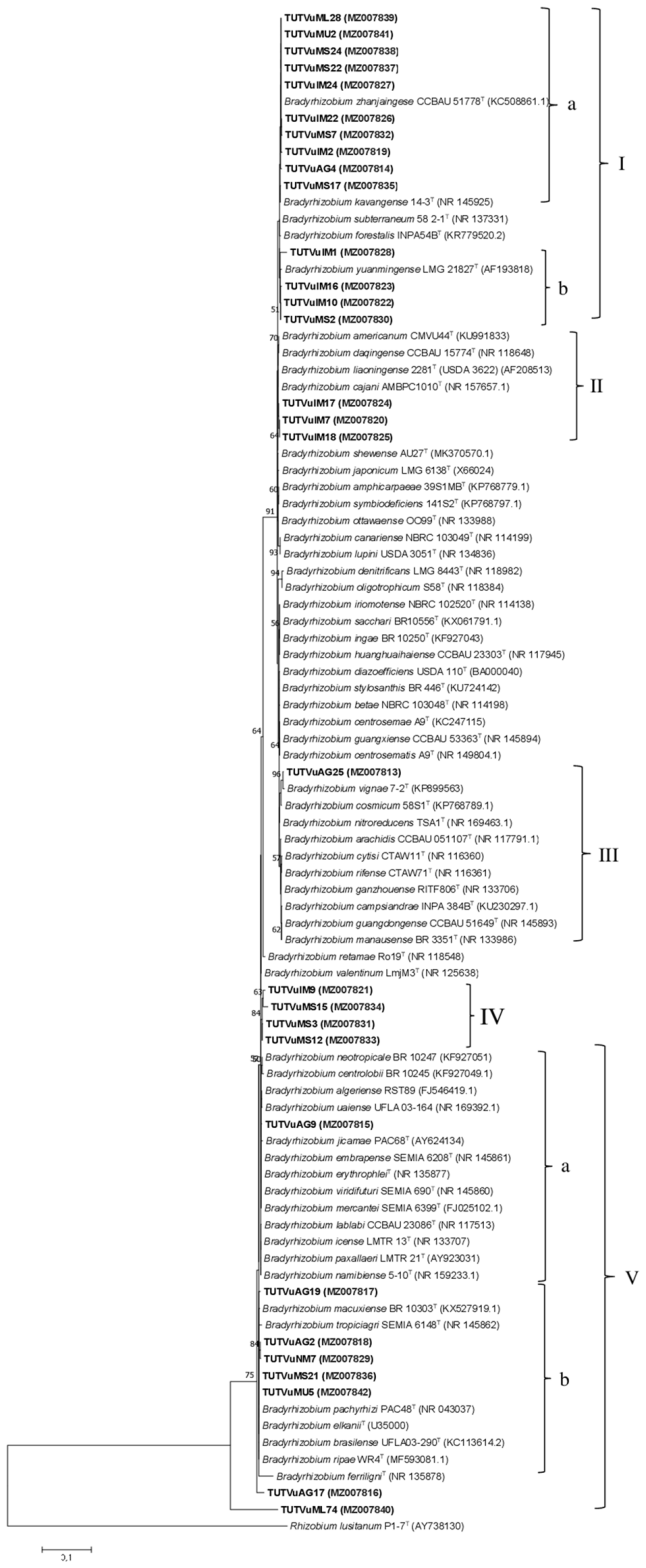
Maximum likelihood molecular phylogenetic analysis of native cowpea nodulating *Bradyrhizobium* isolates from Mozambican soils based on 16S rRNA gene sequences. The aligned sequences were trimmed to a uniform length of 958 bp for the construction of the phylogenetic tree. For each isolate and reference type strain, the accession numbers are indicated in brackets.

**Figure 4:**
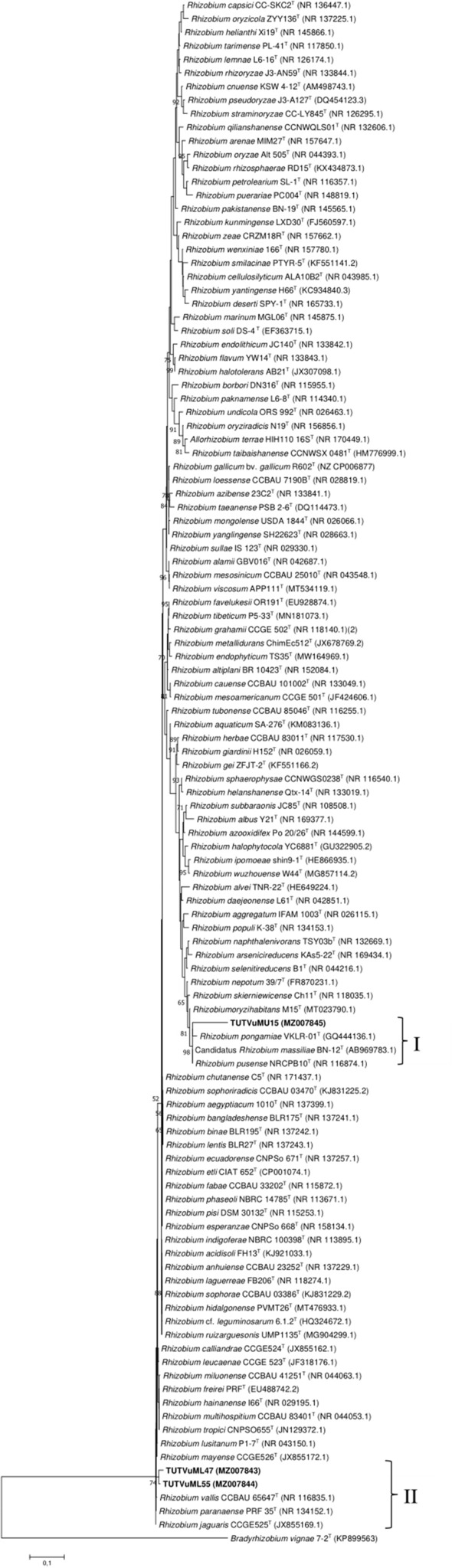
Maximum likelihood molecular phylogenetic analysis of native cowpea nodulating *Rhizobium* isolates from Mozambican soils based on 16S rRNA gene sequences. The aligned sequences were trimmed to a uniform length of 1127 bp for the construction of the phylogenetic tree. For each isolate and reference type strain, the accession numbers are indicated in brackets.

Representative isolates with variable symbiotic effectiveness were selected from the various ERIC-PCR clusters for phylogenetic analysis based on sequence analysis of their 16S rRNA gene, which assigned 30 isolates to species of the genus *Bradyrhizobium* (Fig. 3) and only three isolates to the genus *Rhizobium* (Fig. 4). The aligned sequences were trimmed to a final uniform length of 958 bp for the *Bradyrhizobium* isolates and 1127 bp for the *Rhizobium* isolates before the construction of phylogenetic tress. On the *Bradyrhizobium* branch of the phylogenetic tree, isolates from different locations in the two agroecologies grouped together (with 95.5–100% sequence similarity) and shared 99.6–100% similarity with the type strain *Bradyrhizobium zhanjaingese* in Cluster Ia (Fig. 3). Cluster 1b on the same branch comprised isolates TUTVuMS2 from Mussuril and three other isolates from Ilha de Moçambique which grouped together (98.4–99.8% sequence similarity) and shared 98.2–99.5% sequence similarity with *B. yuanmingense* and *B. cajani* (Fig. 3). Although isolates TUTVuIM7, TUTVuIM17 and TUTVuIM18 stood alone in Cluster II (99.7–99.8% sequence similarity), they shared 99.6–99.8% sequence similarity with the type strains *B. liaoningense* and *B. cajani*. Furthermore, isolate TUTVuAG25 shared 99.5%% similarity with *B. vignae* in Cluster III (Fig. 3). However, the fours isolates in Cluster IV grouped alone, but shared 98.7–99.6% sequence similarity with *B. icense* and *B. paxallaeri*. On the other hand, Cluster V comprised isolates from different locations which shared 99.1–99.7% sequence similarity. Within Cluster V, the isolates shared 99.2–99.6% sequence similarity with type strains such as *B. elkanii, B. pachyrhizi*, *B. icense*, *B. mercantei*, *B. namibiense* and *B. erythrophlei* (Fig. 3). However, isolates TUTVuAG17 and TUTVuML74 stood as out-groups away from any reference type strain (Fig. 3).

On the *Rhizobium* branch of the phylogenetic tree (Fig. 4), isolate TUTVuMU15 grouped with *R. pusense* and *R. massiliae*, with 90.5 and 90.7% sequence similarity, respectively in Cluster I. On the other hand, isolates TUTVuML47 and TUTVuML55 grouped in Cluster II (98.4% sequence similarity) and shared 98.0–98.8% similarity with *R. jaguaris, R. miluonense* and *R. paranaense* (Fig. 4).

### Nodule numbers and dry matter

**Figure 5:**
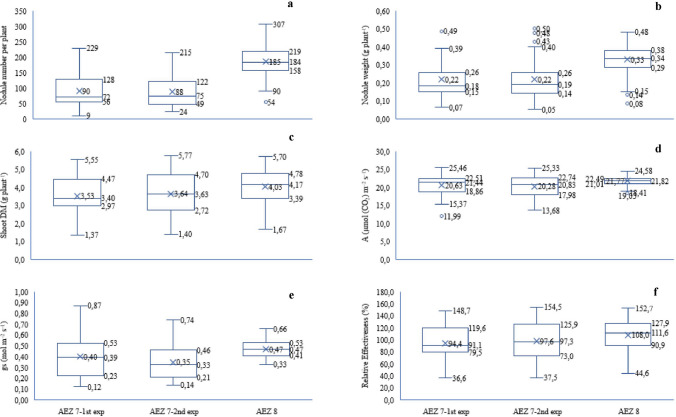
A Box and whisker plot analyses of the variability in nodulation, shoot biomass and photosynthetic physiology of cowpea inoculated with native rhizobial isolates from AEZ 7 and AEZ 8 of the Nampula Province, Mozambique. Centre lines indicate the median; X indicates the mean while box limits indicate the lower and upper quartiles; outliers are indicated by dots.

The 137 rhizobial isolates were tested for symbiotic effectiveness which showed variable nodulation capabilities (Fig. 5; Tables S1 and S2). The variation in nodule number elicited by the isolates from AEZ 7 in Experiments 1 and 2 were nearly similar, with interquartile range of 72 and 73 nodules per plant, respectively (Fig. 5a). In contrast, the number of nodules induced by the isolates from AEZ 8 in a single nodulation assessment was found having less variability with interquartile range of 61, as indicated by the relatively smaller box limits (Fig. 5a). However, the isolates from AEZ 8 generally induced more nodules ranged from 54 nodules per plant by isolate TUTVuMS10 to 307 nodules by TUTVuIM2 (Fig. 5a; Table S2). This contrasted with much lower number produced by isolates from AEZ 7 in Experiments 1 and 2, which ranged from 9 nodules per plant by isolate TUTVuNM8 to 229 nodules by TUTVuML37 (Fig. 5a; Table S1a and S1b). Furthermore, the differences in nodule DM per plant produced by the isolates from the two agroecologies followed a similar trend as their nodule numbers (Fig. 5b). Of the isolates from AEZ 7, 24% from experiment 1 and 26% from experiment 2, formed significantly more nodules (p ≤ 0.05) on cowpea when compared to the commercial *Bradyrhizobium* sp. strain CB756 in both Experiments 1 and 2 (Table S1a and S1b). However, about 88% of the isolates from AEZ 8 formed greater nodule numbers compared with the commercial strain CB756 (Table S2).

### Shoot dry matter

The shoot DM production induced by isolates obtained from AEZ 7 varied, indicating relatively higher interquartile range in experiment 1 (1.5 g plant^−1^) and experiment 2 (2.0 g plant^−1^), when compared to the smaller box limits or interquartile range (1.4 g plant^−1^) of the values induced by the isolates from AEZ 8 (Fig. 5c). Although plant growth assessed by shoot biomass elicited by the isolates from AEZ 8 showed less variability in this aspect, the values were generally greater. They ranged from a minimum of 1.67 g plant^−1^ induced by isolate TUTVuIM3 to 5.70 g plant^−1^ by TUTVuMS25 (Fig. 5c; Table S2). In contrast, the isolates from AEZ 7 expressed less plant growth, with shoot biomass ranging from 1.37 to 5.77 g plant^−1^ by isolates TUTVuNM10 and TUTVUML27, respectively, and with a mean of 3.53 g plant^−1^ in experiment 1 and 3.64 g plant^−1^ in experiment 2 (Fig. 5c; Table S1a and S1b).

Of the isolates from AEZ 7, 9 out of the 33 tested in Experiment 1 and 22 out of the 46 isolates in Experiment 2 induced greater (p ≤ 0.05) shoot DM accumulation than the commercial *Bradyrhizobium* strain CB756 which recorded shoot DM of 2.94 and 3.10 g plant^−1^, respectively (Table S1a and S1b). Of the same AEZ 7 isolates, 6 from Experiment 1 and 9 from Experiment 2 induced significantly greater plant growth (shoot biomass) than the 5 mM KNO_3_ fertilization (Table S1a and S1b). Moreover, 10 out of the 58 isolates from AEZ 8 induced significantly greater shoot DM accumulation than the application of 5 mM nitrate (Table S2). On the other hand, 27% of the 58 isolates from AEZ 8 stimulated higher nodule DM than the commercial strain CB756 (Table S2).

### Photosynthesis and stomatal conductance

There was marked variability in the photosynthetic rates and stomatal conductance elicited by the tested isolates from the two agroecological districts, which came in accordance with the observed differences in root nodulation and shoot DM (Fig. 5a–d).

Of the isolates from AEZ 7, 7 isolates in experiment 1 and only one isolate in experiment 2 elicited greater photosynthetic rates than what was induced by the commercial strain CB756, while 25 and 24, respectively, induced higher photosynthetic rates than N-fed plants (Table S1a and S1b). However, the commercial *Bradyrhizobium* sp. strain CB756 together with 42 of the tested isolates from AEZ 8 induced greater photosynthetic rates (p < 0.05) than 5 mM in nitrate-feeding (Table S2).

### Relative effectiveness of rhizobial isolates

**Figure 6:**
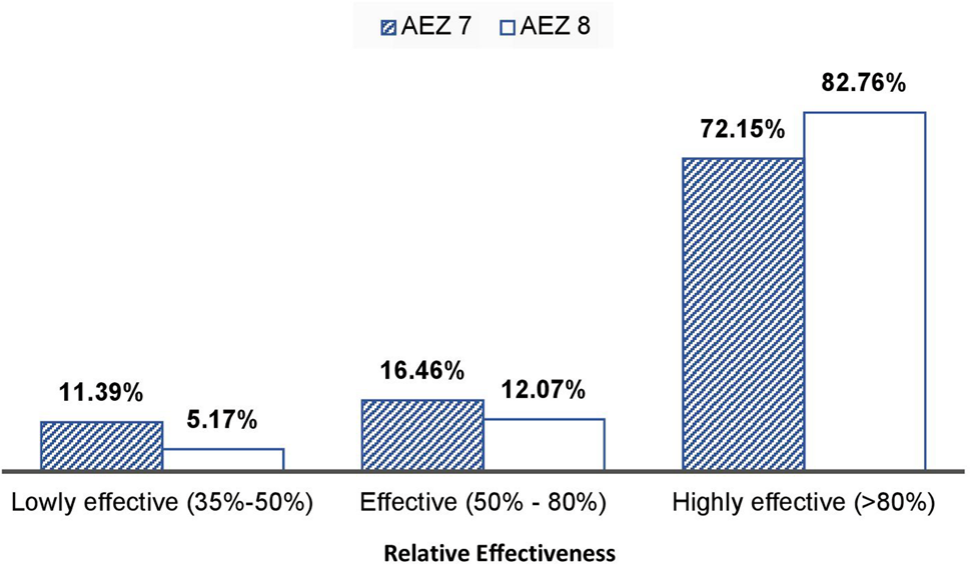
Summary of relative effectiveness of authenticated rhizobial isolates from agroecological zones 7 and 8 in Mozambique.

The relative effectiveness of the tested isolates, based on host plant shoot DM, expectedly showed similar variation as the plant growth induced by the isolates from the two agroecologies. The isolates from AEZ 7 exhibited marked variation in their relative effectiveness, with values ranged from 37% for isolate TUTVuNM10 to 155% for TUTVuML27, with a mean of 94% in experiment 1 and 98% in experiment 2 (Fig. 5f). Despite the relatively low variability in the relative effectiveness of isolates from AEZ 8, they were generally more effective as they expressed a higher mean relative effectiveness with values which ranged from 45% for isolate TUTVuIM3 to 153% for the TUTVuMS25 with a mean of 108% (Fig. 5f). When all the isolates from the two agroecologies were compared, 11% from AEZ 7 and 5% from AEZ 8 exhibited low effectiveness of 35–50% RE, 16% from AEZ 7 and 12% from AEZ 8 were effective (50–80% RE), while 72% from AEZ 7 and 83% from AEZ 8 were highly effective (> 80% RE) (Fig. 6; Table S1a, S1b and S2).

### Comparing the symbiotic parameters of the most highly effective rhizobial isolates from AEZ 7 and AEZ 8

For brevity, the top 25% of the high N_2_-fixing isolates in the upper whiskers of the box plots for the two agroecologies (Fig. 5f) were compared in Table 2. A one-way ANOVA revealed significant differences in nodule number, nodule DM, photosynthetic rates and stomatal conductance induced by the top N_2_-fixing isolates from the two agroecologies (Table 2). For example, inoculating cowpea with isolates TUTVUIM2, TUTVuIM17, TUTVuAG11 and TUTVuIM22 from AEZ 8 resulted in higher (p ≤ 0.05) nodule numbers of 307 ± 14, 288 ± 40, 286 ± 32 and 239 ± 12 /plant, respectively, than the isolates from AEZ 7 that were used in experiments 1 and 2 which produced 58 to 193 nodules/plant (Table 2). The four isolates also elicited more nodules on the homologous cowpea host than the commercial *Bradyrhizobium* sp. strain CB756 which produced 130 ± 6 and 119 ± 8 nodules in experiments 1 and 2 with isolates from AEZ 7 and 123 ± 11 nodules when tested with isolates from AEZ 8 (Table 2). On the other hand, isolates TUTVuML55, TUTVuML63, TUTML41, TUTVuML75, TUTVuMU13 and TUTVuMU4 from AEZ 7 induced much higher nodule DM on cowpea (values ranged from 0.40 to 0.50 g plant^−1^) than most isolates from AEZ 8 (which produced relatively more nodules) and the commercial inoculant strain CB756 which recorded 0.19 and 0.21 g plant^−1^ nodule DM in experiments 1 and 2, respectively) (Table S1).

**Table 2:** Nodulation, shoot biomass, relative effectiveness and photosynthetic functioning of cowpea inoculated with top 25% of highly effective rhizobia isolated from cowpea root nodules sampled from AEZ 7 and AEZ8 of the Nampula Province, Mozambique.

Origen of isolates	Treatment	Nodule number	Nodule dry weight	Shoot dry matter	A	gs	Relative effectiveness
		no. plant^−1^	g plant^−1^	g plant^−1^	μmol (CO_2_) m^–2^ s^–1^	mol m^–2^ s^–1^	%
	**Isolate**						
AEZ 7 1st Experiment	TUTVuML41	155.00 ± 8.00c-j	0.49 ± 0.01ab	5.55 ± 0.55a	16.91 ± 0.10nop	0.18 ± 0.01nop	148.66 ± 14.69a
	TUTVuML74	83.00 ± 35.00k–n	0.32 ± 0.04d–i	5.33 ± 0.43ab	18.60 ± 0.21k–n	0.16 ± 0.01nop	142.86 ± 11.40ab
	TUTVuML55	142.00 ± 21.00e–m	0.50 ± 0.06ab	5.33 ± 0.67ab	22.17 ± 0.91b–f	0.45 ± 0.01c–i	142.86 ± 17.92ab
	TUTVuML75	125.00 ± 12.00f–n	0.49 ± 0.04ab	5.28 ± 0.22ab	17.96 ± 0.01l–o	0.19 ± 0.00nop	141.29 ± 5.80ab
	TUTVuML16	131.00 ± 44.00f–n	0.39 ± 0.07a–f	5.03 ± 0.38ab	19.11 ± 0.26j–m	0.23 ± 0.01l–o	134.82 ± 10.30ab
	TUTVuML12	58.00 ± 14.00n	0.20 ± 0.01k–n	4.90 ± 0.12abc	22.16 ± 0.01b–f	0.56 ± 0.00ab	131.25 ± 3.09abc
	TUTVuML66	196.00 ± 23.00c–f	0.34 ± 0.06d–h	4.67 ± 0.93a–e	11.99 ± 0.15r	0.12 ± 0.00pq	125.00 ± 25.00abc
	TUTVuML18	72.00 ± 2.00mn	0.35 ± 0.01d–h	4.63 ± 0.78a–e	20.81 ± 0.84g–j	0.38 ± 0.02h–k	124.11 ± 20.77abc
	TUTVuMU10	61.00 ± 7.00n	0.14 ± 0.02n	4.30 ± 0.64b–f	25.46 ± 0.42a	0.54 ± 0.04abc	115.18 ± 17.22bcd
AEZ 7 2nd Experiment	TUTVuML11	193.00 ± 43.00c–g	0.37 ± 0.02c–g	5.73 ± 0.20a	17.23 ± 0.76mno	0.17 ± 0.01nop	153.57 ± 5.43a
	TUTVuML27	147.00 ± 37.00d–k	0.48 ± 0.08abc	5.77 ± 0.33a	20.82 ± 1.38g–j	0.40 ± 0.06g–k	154.46 ± 8.93a
	TUTVuML63	122.00 ± 8.00f–n	0.50 ± 0.02a	5.75 ± 0.26a	23.36 ± 0.09b	0.31 ± 0.01klm	154.02 ± 6.96a
	TUTVuMU13	122.00 ± 11.00f–n	0.43 ± 0.03a–d	5.53 ± 0.27a	13.68 ± 0.00pr	0.14 ± 0.00opq	148.21 ± 7.31ab
	TUTVuML3	58.00 ± 15.00n	0.20 ± 0.05k–n	5.40 ± 0.06ab	18.06 ± 0.35k–n	0.22 ± 0.02m–p	144.64 ± 1.55ab
	TUTVuML25	122.00 ± 12.00f–n	0.31 ± 0.07e–k	5.33 ± 0.82ab	20.02 ± 0.13h–k	0.16 ± 0.01nop	142.86 ± 22.00ab
	TUTVuML19	90.00 ± 4.00j–n	0.32 ± 0.02d–j	5.27 ± 0.55ab	19.39 ± 0.71i–l	0.19 ± 0.01nop	141.07 ± 14.70ab
	TUTVuML47	174.00 ± 53.00c–h	0.36 ± 0.04c–h	5.17 ± 0.59ab	18.25 ± 2.49k–n	0.20 ± 0.04nop	138.39 ± 15.87ab
	TUTVuMU4	75.00 ± 11.00lmn	0.40 ± 0.06a–e	5.00 ± 0.85ab	18.24 ± 0.75k–n	0.24 ± 0.04l–o	133.93 ± 22.78ab
	TUTVuML28	175.00 ± 18.00c–h	0.19 ± 0.01lmn	4.77 ± 0.15a–d	22.22 ± 0.85b–f	0.39 ± 0.03g–k	127.68 ± 3.89abc
	TUTVuML30	93.00 ± 9.00i–n	0.21 ± 0.04j–n	4.70 ± 0.51a–d	22.41 ± 0.77b–f	0.33 ± 0.02jkl	125.89 ± 13.75abc
	TUTVuML31	116.00 ± 9.00h–n	0.25 ± 0.01h–m	4.70 ± 0.10a–d	15.26 ± 1.23pq	0.14 ± 0.02opq	125.89 ± 2.68abc
	**Isolate**						
AEZ 8	TUTVuMS25	171.00 ± 2.00c–h	0.35 ± 0.02d–h	5.70 ± 0.21a	20.97 ± 0.37g–j	0.37 ± 0.01h–k	152.69 ± 5.58a
	TUTVuAG11	286.00 ± 52.00ab	0.34 ± 0.05d–h	5.57 ± 0.52a	21.78 ± 0.99b–g	0.43 ± 0.08d–i	149.12 ± 13.95a
	TUTVuMS24	158.00 ± 46.00c–j	0.27 ± 0.04g–m	5.57 ± 0.03a	20.62 ± 0.40g–j	0.37 ± 0.03h–k	149.12 ± 0.89a
	TUTVuAG17	168.00 ± 9.00c–h	0.41 ± 0.03a–e	5.37 ± 0.15ab	21.36 ± 0.54c–g	0.38 ± 0.05h–k	143.76 ± 3.89ab
	TUTVuMS2	146.00 ± 11.00d–l	0.30 ± 0.03e–l	5.30 ± 0.40ab	21.77 ± 0.75b–g	0.46 ± 0.06c–i	141.98 ± 10.83ab
	TUTVuAG19	219.00 ± 57.00b–d	0.39 ± 0.03b–g	5.23 ± 0.62ab	22.93 ± 0.76b–e	0.36 ± 0.03ijk	140.19 ± 16.68ab
	TUTVuIM2	307.00 ± 14.00a	0.34 ± 0.07d–h	5.20 ± 0.45ab	21.92 ± 0.71b–g	0.53 ± 0.05a–d	139.30 ± 12.08ab
	TUTVuMS22	165.00 ± 17.00c–i	0.28 ± 0.05f–m	5.17 ± 0.32ab	22.03 ± 0.46b–f	0.39 ± 0.04g–k	138.41 ± 8.52ab
	TUTVuAG4	225.00 ± 34.00bcd	0.35 ± 0.05d–h	5.13 ± 0.37ab	22.04 ± 0.70b–f	0.50 ± 0.03b–f	137.51 ± 9.94ab
	TUTVuIM22	239.00 ± 12.00abc	0.40 ± 0.01a–e	5.13 ± 0.19ab	23.08 ± 0.66bcd	0.56 ± 0.05ab	137.51 ± 4.97ab
	TUTVuMS17	218.00 ± 44.00b–d	0.41 ± 0.06a–e	5.10 ± 0.36ab	22.41 ± 0.44b–f	0.48 ± 0.01b–g	136.62 ± 9.66ab
	TUTVuIM18	226.00 ± 39.00bcd	0.39 ± 0.01a–f	5.00 ± 0.25ab	22.08 ± 0.51b–f	0.51 ± 0.06b–e	133.94 ± 6.74ab
	TUTVuIM17	288.00 ± 40.00ab	0.42 ± 0.03a–d	4.87 ± 0.46abc	21.00 ± 1.31f–i	0.47 ± 0.13b–h	130.37 ± 12.41abc
	TUTVuIM24	207.00 ± 25.00c–e	0.30 ± 0.05e–l	4.80 ± 0.10a–d	21.25 ± 0.55d–h	0.45 ± 0.04c–i	128.58 ± 2.68abc
	TUTVuAG1	168.00 ± 3.00c–i	0.43 ± 0.02a–d	4.77 ± 0.32a–d	21.10 ± 0.25f–h	0.41 ± 0.02f–k	127.69 ± 8.52abc
*B.* strain CB756 ^‡^		130.00 ± 0.00f–n	0.19 ± 0.00mn	2.94 ± 0.00f	20.58 ± 0.32g–j	0.42 ± 0.00e–j	88.39 ± 3.09d
*B.* strain CB756 ^‡‡^		119.00 ± 0.00g–n	0.21 ± 0.00k–n	3.10 ± 0.00f	22.50 ± 0.01b–f	0.56 ± 0.00ab	88.39 ± 3.09d
*B.* strain CB756 ^‡‡‡f^		123.00 ± 1.00f–n	0.22 ± 0.00i–n	3.30 ± 0.12fg	23.22 ± 0.42bc	0.62 ± 0.03a	88.40 ± 3.09d
5 mM KNO_3_^‡^		–	–	3.51 ± 0.11efg	16.10 ± 0.01op	0.25 ± 0.00lmn	100.00 ± 11.61cd
5 mM KNO_3_^‡‡^		–	–	3.68 ± 0.06d–g	16.92 ± 0.00nop	0.23 ± 0.00l–o	100.00 ± 11.61cd
5 mM KNO_3_^‡‡‡^		–	–	3.73 ± 0.43c–g	19.08 ± 0.36j–m	0.40 ± 0.00g–k	100.01 ± 11.61cd
Uninoculated^‡^		–	–	0.43 ± 0.01g	0.59 ± 0.00s	0.02 ± 0.00r	–
Uninoculated^‡‡^		–	–	0.45 ± 0.00g	0.63 ± 0.01s	0.02 ± 0.00r	–
Uninoculated^‡‡‡^		–	–	0.48 ± 0.04g	0.88 ± 0.03s	0.05 ± 0.00qr	–
*F* statistics		5.75***	5.94***	10.17***	64.61***	19.81***	2.37***

Values (Means ± SE) with dissimilar letters in a column are significant at ***p ≤ 0.001. ^‡^ and ^‡‡^: Controls on Experiments 1 and 2 with isolates from AEZ 7 and ^‡‡‡^ controls on Experiment with isolates from AEZ 8.

Of the top N_2_-fixing isolates selected from those of AEZ 7, TUTVuMU10 and TUTVuML63 from Muriase and Mulapane elicited the highest photosynthetic rates of 25.46 and 23.36 μmol CO_2_ m^−2^ s^−1^, respectively than the remaining isolates from AEZ 7 and AEZ 8 as well as the commercial inoculant strain CB756 (Table 2; Table S1). In general, the isolates that expressed greater stomatal conductance also stimulated high photosynthetic activities. However, there were some exceptions (Table 2).

The top 25% of the rhizobial isolates tested from AEZ 7 and AEZ 8 produced nearly similar shoot DM yield which ranged from 4.63 to 5.77 g plant^−1^, except for TUTVuMU10 which scored only 4.30 g plant^−1^ (Table 2). The relative effectiveness values of the selected isolates ranged from 115 to 154%. The 5 mM KNO_3_ supplemented plants were considered to have 100% theoretical RE while the plants inoculated with strain CB756 scored 88.4% RE, in comparison (Table 2).

### Correlation analysis

The correlation analysis performed for nodule number, nodule dry weight and shoot dry matter induced by rhizobial isolates collected from AEZ 7 and AEZ 8 revealed significantly positive relationships between nodule number and nodule dry weight at AEZ 7 for experiments 1 and 2 (r = 0.6328*** and r = 0.6431***, respectively), and at AEZ 8 (r = 0.5801***). Nodule number and shoot dry matter were significantly correlated at AEZ 7 for both the experiments 1 and 2, calculated to be r = 0.4018*** and r = 0.4723***, respectively, and at AEZ 8 (r = 0.3543***). Same was found for nodule dry weight and shoot dry matter with isolates from AEZ 7 when used in experiments 1 and 2 (r = 0.6349*** and r = 0.6071***, respectively), while in AEZ 8 the correlation was r = 0.2996*** (Table S3).

## Discussion

### Morpho-genetic diversity of rhizobial microsymbionts of cowpea in Mozambique

Cowpea rhizobial strains are widely distributed in soils across Africa and other parts of the world^38–42^. They exhibit marked morpho-genomic variability^35,43,44^. In this work, of a total of 203 cowpea root-nodule bacteria isolated, 175 were able to elicit functional nodules on their homologous host. Most of the isolates that failed to nodulate the host plant are most likely non-nodulating endophytes resident in nodules^35^. Additionally, these isolated bacterial symbionts of cowpea which were collected from two agroecologies of Mozambique exhibited marked morphological variability as evidenced by differences in colony size, shape, texture, colour and growth rate (Table 1). The majority of them were slow-growers with small colony sizes, a finding consistent with an earlier report that found that most cowpea symbionts in Mozambican soils form smaller colonies with diameters less than 1.5 mm^35^. Mohammed et al.^44^ also isolated slow-growing cowpea rhizobia from Ghanaian and South African soils that had small colony sizes as well as similar colour and shape as those found in this study. Interestingly, the rhizobial isolates collected for this study were found to be genetically diverse and could be grouped into 157 distinct ERIC-PCR types at a 70% similarity cut-off point (Fig. 2), which is consistent with other studies that found cowpea can be nodulated by genetically diverse rhizobial populations in Mozambique and other African countries^39,43,44,54,55^. Using one cowpea genotype, Namuruwa, as a trap host in AEZ 8 and several other genotypes IT-16, IT-18, Sudan-1, T-1263 and T99K-529-1 as well as Namuruwa used in AEZ 7 did not influence the diversity of the isolates from AEZ 8, suggesting that there was no effect of the host cultivars on isolate distribution in the ERIC-PCR clusters (Fig. 2). Interestingly, no rhizobial isolates from any of the cowpea genotypes used formed an exclusive cluster, confirming recent reports that cultivar differences had no influence on the genomic relatedness of rhizobial symbionts nodulating cowpea and Kersting’s groundnut in Ghanaian, South African and Mozambican soils^44,56^. Furthermore, the fact that some ERIC-PCR clusters were homogenous in terms of the origin of isolates, and others heterogenous (comprising isolates from different agroecologies) suggests that, while the distribution of rhizobial types may be shaped by edaphic and climatic factors, some others seem to exhibit wider adaptation and could therefore occur in contrasting environments. This was also reported by Mohammed et al.^44^, who showed that some bacterial symbionts of cowpea from contrasting locations in Ghana and South Africa shared closer genomic relatedness.

To identify the rhizobia responsible for cowpea nodulation in the agroecologies studied, representative isolates were subjected to phylogenetic analysis based on sequence analysis of their 16S rRNA genes. Of the 33 isolates selected from the major ERIC-PCR clusters, the phylogenetic analysis aligned 30 isolates to species belonging to the genus *Bradyrhizobium* while three isolates showed closer relations with species in the genus *Rhizobium*. Thus, whereas some isolates in this study closely aligned with *B. zhanjaingese*, *B. yuanmingense*, *B. vignae* and *B. elkanii* in the *Bradyrhizobium* branch of the phylogenetic analysis (Fig. 3), other isolates, including TUTVuML47 and TUTVuML55, showed closer alignment with *R. jaguaris, R. miluonense* and *R. paranaense* as evidenced in the *Rhizobium* branch in Fig. 4. However, our findings are in accordance with an earlier report by Chidebe et al.^35^ who found that the majority of cowpea symbionts in Mozambique are aligned to species of the genus *Bradyrhizobium*, while fewer isolates were found aligned with species in the genus *Rhizobium*. The bradyrhizobial symbionts identified in this study included highly divergent isolates such as TUTVuAG17 and TUTVuML74 that can potentially represent novel species, pointing Africa as a hotspot for bradyrhizobial diversity, as also reported earlier^37,44,57^.

### Symbiotic and photosynthetic functions

This study revealed high variability in the symbiotic effectiveness and photosynthetic functioning parameters induced by inoculation with the native rhizobial symbionts of cowpea obtained from the two contrasting agroecologies in Mozambique. Although the isolates from AEZ 7 induced more variable nodulation capacities, their counterparts from AEZ 8 induced greater nodulation with a lower quartile that was much greater than the upper quartiles of the nodule number and nodule DM produced by isolates from AEZ 7 (Fig. 5). The observed variation in cowpea nodulation by the tested isolates closely mirrored the photosynthetic functioning they induced in the homologous host; however, the isolates from AEZ 8 stimulated greater photosynthesis and stomatal conductance which were less variable when compared to values induced by the isolates from AEZ 7. The fact that increased nodulation and photosynthetic rates caused by isolates from AEZ 8 led to higher but relatively less variable shoot biomass and relative symbiotic efficiency compared to the isolates from AEZ 7, suggests that the greater nodulation did not always translate into higher N_2_ fixation and/or plant growth. This strongly suggests that some nodule occupants were less effective in fixing nitrogen. A number of studies have shown that legumes sometimes form ineffective and inefficient root nodules with indigenous rhizobia^58,59^. In this study, however, the isolates from AEZ 8 were far more effective and capable of inducing greater shoot biomass with the lower quartile of their shoot DM and/or relative effectiveness values being closer or similar to the median of the values for those parameters induced by the isolates from AEZ 7, as shown in Fig. 5. The strong variation in the photosynthetic efficiency and symbiotic effectiveness of the indigenous isolates in this study emphasises the need to screen for high N_2_-fixing and locally well adapted isolates for inoculant formulations.

The shoot dry matter of legumes raised in a N-free media can be considered a good indicator of the symbiotic effectiveness of their rhizobial symbionts^56^. As the uninoculated control plants expectedly exhibited the least plant growth, the effectiveness of isolates was assessed by comparing the biomass of inoculated plants with that of nitrate fed plants^37^. Regarding the marked differences in the relative effectiveness of the isolates used in this study, 58 out of the 79 isolates from AEZ 7 and 48 out of the 58 isolates from AEZ 8 were scored as highly effective, with > 80% RE. These results suggest the presence of highly effective rhizobial populations capable of cowpea nodulation in Mozambican soils, which came in accordance with Mohammed et al.^44^ who isolated very effective native rhizobia from root nodules of cowpea grown in Ghana and South Africa. Conclusively, there are many super effective rhizobia in African soils that are waiting to be discovered for use in agriculture!

In this study, although the top 25% of high N_2_-fixing isolates differed markedly in numbers and biomass of the nodules they induced, the differences in the shoot biomass produced was statistically not-significant for most isolates, thus, confirming that plant nodulation per se is not a sole indicator of symbiotic performance. However, these isolates generally supported better plant growth than the commercial inoculant of *Bradyrhizobium* sp. strain CB756 and quantity of the applied mineral N used. However, the greater photosynthetic functioning elicited in cowpea by the top N_2_-fixing isolates obtained from the two agroecologies can be related to increases in synthesis of chlorophyll in the inoculated plants, as compared to the nitrate feeding counterparts, and/or enhanced biosynthesis of Rubisco, the enzyme needed for photosynthesis^49^. Several studies have shown that rhizobial inoculation can enhance symbiotic N supply, increase leaf chlorophyll and photosynthetic rates^36,44,56,60^. The observed variability in photosynthetic rates and leaf stomatal conductance was found much greater for the isolates from AEZ 7 when compared to the values of those parameters induced by isolates from AEZ 8. For example, the photosynthetic rates and stomatal conductance induced by isolates from AEZ 7 had a greater interquartile range, as indicated by their wider box limits relative to their counterparts from AEZ 8 which had narrower box limits (Fig. 5d and e). Furthermore, the lower quartile of the photosynthetic rates and stomatal conductance induced by the isolates from AEZ 8 was either greater or closer to the median values of the same parameters produced by the isolates from AEZ 7 in both the Experiments 1 and 2 (Fig. 5d and e).

## Conclusion

The study here revealed the presence of highly effective rhizobia in Mozambican soils that can be exploited for inoculant production as an alternative to the use of expensive fossil-based chemical fertilizers. Furthermore, the high population of indigenous cowpea microsymbionts, and probably those of other legumes, from contrasting agroecologies of Mozambique exhibited high N_2_-fixation efficiency and can elicit greater photosynthetic rates in cowpea than N-fertilization. Their potential for use in inoculant formulations should be considered for enhanced crop production, reduced input costs and assistance of environmental soundness.

## Data availability:

Datasets used in this study are available from the corresponding author on reasonable request.

## Supplementary Information

Supplementary Information.

## References

[1] Hörtensteiner S, Feller U (2002). Nitrogen metabolism and remobilization during senescence. J. Exp. Bot..

[2] Danku JMC, Lahner B, Yakubova E, Salt DE (2013). Plant Mineral Nutrients Methods and Protocols.

[3] Hokmalipour S, Darbandi MH (2012). Effects of nitrogen fertilizer on chlorophyll content and other leaf indicate in three cultivars of maize (*Zea mays* L.). World Appl. Sci. J..

[4] Tairo E, Mtei K, Ndakidemi P (2017). Influence of water stress and rhizobial inoculation on the accumulation of chlorophyll in *Phaseolus vulgaris* (L.) Cultivars. Int. J. Plant Soil Sci..

[5] Gai Z, Zhang J, Li C (2017). Effects of starter nitrogen fertilizer on soybean root activity, leaf photosynthesis and grain yield. PLoS ONE.

[6] Mmbaga GW, Mtei KM, Ndakidemi PA (2014). Extrapolations on the use of *Rhizobium* inoculants supplemented with Phosphorus (P) and Potassium (K) on growth and nutrition of legumes. Agric. Sci..

[7] Zhong C, Cao X, Hu J, Zhu L, Zhang J (2017). Nitrogen metabolism in adaptation of photosynthesis to water stress in rice grown under different nitrogen levels. Front. Plant Sci..

[8] Cao X (2017). Effects of watering regime and nitrogen application rate on the photosynthetic parameters, physiological characteristics, and agronomic traits of rice. Acta Physiol. Plant..

[9] Drechsel P, Gyiele L, Kunze D, Cofie O (2001). Population density, soil nutrient depletion, and economic growth in sub-Saharan Africa. Ecol. Econ..

[10] Sanchez PA (2002). Soil fertility and hunger in Africa. Sci. Compass.

[11] Mafongoya PL, Bationo A, Kihara J, Waswa BS (2006). Appropriate technologies to replenish soil fertility in southern Africa. Nutr. Cycl. Agroecosyst..

[12] Chianu JN, Chianu JN, Mairura F (2012). Mineral fertilizers in the farming systems of sub-Saharan Africa. A review. Agron. Sustain. Dev..

[13] Morris M, Kelly V, Kopicki R, Byerlee D (2007). Fertilizer Use in African Agriculture: Lessons Learned and Good Practice Guidelines.

[14] Maaso C (2007). Dilemma of nitrogen management for future food security in sub-Saharan Africa: A review Review Cargele. Soil Res..

[15] Lengwati D, Mathews C, Dakora F (2020). Rotation benefits from N_2_-fixing grain legumes to cereals: From increases in seed yield and quality to greater household cash-income by a following maize crop. Front. Sustain. Food Syst..

[16] Unkovich M (2008). Measuring Plant-Associated Nitrogen Fixation in Agricultural Systems.

[17] Dakora FD, Keya SO (1997). Contribution of legume nitrogen fixation to sustainable Agriculture in Sub-Saharan Africa. Soil Biol. Biochem..

[18] Herridge DF, Peoples MB, Boddey RM (2008). Global inputs of biological nitrogen fixation in agricultural systems. Plant Soil.

[19] Chikowo R, Mapfumo P, Nyamugafata P, Giller KE (2004). Woody legume fallow productivity, biological N_2_-fixation and residual benefits to two successive maize crops in Zimbabwe. Plant Soil.

[20] Pule-Meulenberg F, Gyogluu C, Naab J, Dakora FD (2011). Symbiotic N nutrition, bradyrhizobial biodiversity and photosynthetic functioning of six inoculated promiscuous-nodulating soybean genotypes. J. Plant Physiol..

[21] Naab JB, Chimphango SMB, Dakora FD (2009). N_2_ fixation in cowpea plants grown in farmers’ fields in the Upper West Region of Ghana, measured using ^15^N natural abundance. Symbiosis.

[22] Odendo M, Bationo A, Kimani S (2011). Socio-economic Contribution of Legumes to Livelihoods in Sub-Saharan Africa, Fighting Poverty in Sub-Saharan Africa: The Multiple Roles of Legumes in Integrated Soil Fertility Management.

[23] Dakora FD, Aboyinga RA, Mahama Y, Apaseku J (1987). Assessment of N_2_ fixation in groundnut (*Arachis hypogaea* L.) and cowpea (*Vigna unguiculata* L. Walp) and their relative N contribution to a succeeding maize crop in Northern Ghana. Mircen J. Appl. Microbiol. Biotechnol..

[24] Nyemba RC, Dakora FD (2010). Evaluating N_2_ fixation by food grain legumes in farmers’ fields in three agro-ecological zones of Zambia, using ^15^N natural abundance. Biol. Fertil. Soils.

[25] Bado BV, Bationo A, Cescas MP (2006). Assessment of cowpea and groundnut contributions to soil fertility and succeeding sorghum yields in the Guinean savannah zone of Burkina Faso (West Africa). Biol. Fertil. Soils.

[26] Giller KE (2001). Nitrogen Fixation in Tropical Cropping Systems.

[27] Zahran HH (1999). *Rhizobium*-legume symbiosis and nitrogen fixation under severe conditions and in an arid climate. Microbiol. Mol. Biol. Rev..

[28] Hungria M, Vargas MATT (2000). Environmental factors affecting N_2_ fixation in grain legumes in the tropics, with an emphasis on Brazil. Food Crop. Res..

[29] Mohammed M, Jaiswal SK, Sowley ENKK, Ahiabor BDKK, Dakora FD (2018). Symbiotic N_2_ fixation and grain yield of endangered Kersting’s groundnut landraces in response to soil and plant associated *Bradyrhizobium* inoculation to promote ecological resource-use efficiency. Front. Microbiol..

[30] Chemining’wa GN, Vessey JK (2006). The abundance and efficacy of *Rhizobium leguminosarum* bv. viciae in cultivated soils of the eastern Canadian prairie. Soil Biol. Biochem..

[31] Nyoki D, Ndakidemi P (2013). Economic benefits of Bradyrhizobium japonicum inoculation and phosphorus supplementation in cowpea (*Vigna unguiculata* (L.) Walp) grown in northern Tanzania. Am. J. Res. Commun..

[32] Ulzen J, Abaidoo RC, Mensah NE, Masso C, Abdel Gadir AH (2016). *Bradyrhizobium* inoculants enhance grain yields of soybean and cowpea in Northern Ghana. Front. Plant Sci..

[33] Howieson JG, Malden J, Yates RJ, O’Hara GW (2000). Techniques for the selection and development of elite inoculant strains of *Rhizobium leguminosarum* in southern Australia. Symbiosis.

[34] Catroux G, Hartmann A, Revellin C (2001). Trends in rhizobial inoculant production and use. Plant Soil.

[35] Chidebe IN, Jaiswal SK, Dakora FD (2017). Distribution and phylogeny of microsymbionts associated with cowpea (*Vigna unguiculata* L .Walp.) nodulation in three agroecological regions of Mozambique. Appl. Environ. Microbiol..

[36] Gyogluu C, Mohammed M, Jaiswal SK, Kyei-Boahen S, Dakora FD (2018). Assessing host range, symbiotic effectiveness, and photosynthetic rates induced by native soybean rhizobia isolated from Mozambican and South African soils. Symbiosis.

[37] Chibeba MA, Kyei-boahen S, Guimarães MDF, Nogueira AM, Hungria M (2017). Isolation, characterization and selection of indigenous Bradyrhizobium strains with outstanding symbiotic performance to increase soybean yields in Mozambique. Agric. Ecosyst. Environ..

[38] Law IJ, Botha WF, Majaule UC, Phalane FL (2007). Symbiotic and genomic diversity of ‘cowpea’ bradyrhizobia from soils in Botswana and South Africa. Biol. Fertil. Soils.

[39] Pule-Meulenberg F, Belane AK, Krasova-Wade T, Dakora FD (2010). Symbiotic functioning and bradyrhizobial biodiversity of cowpea (*Vigna unguiculata* L. Walp.) in Africa. BMC Microbiol..

[40] Krasova-Wade T (2003). Diversity of indigeneous bradyrhizobia associated with three cowpea cultivars (*Vigna unguiculata* (L.) Walp.) grown under limited and favorable water conditions in Senegal (West Africa). Afr. J. Biotechnol..

[41] Zhang WT, Yang JK, Yuan TY, Zhou JC (2007). Genetic diversity and phylogeny of indigenous rhizobia from cowpea [*Vigna unguiculata* (L.) Walp.]. Biol. Fertil. Soils.

[42] Zilli JÉ, Valisheski RR, Freire Filho FR, Neves MCP, Rumjanek NG (2004). Assessment of cowpea rhizobium diversity in cerrado areas of Northeastern Brazil. Braz. J. Microbiol..

[43] Dabo M, Jaiswal SK, Dakora FD (2019). Phylogenetic evidence of allopatric speciation of bradyrhizobia nodulating cowpea (*Vigna unguiculata* L. Walp) in South African and Mozambican soils. FEMS Microbiol. Ecol..

[44] Mohammed M, Jaiswal SK, Dakora FD (2018). Distribution and correlation between phylogeny and functional traits of cowpea (*Vigna unguiculata* L. Walp.)-nodulating microsymbionts from Ghana and South Africa. Sci. Rep..

[45] Somasegaran P, Hoben H (1994). Handbook for Rhizobia: Methods in Legume-Rhizobium Technology.

[46] Broughton BWJ, Dilworth MJ (1971). Control of leghaemoglobin synthesis in Snake beans. Biochem. J..

[47] Engels JMM, Dempewolf H, Henson-apollonio V (2011). Ethical considerations in agro-biodiversity research, collecting, and use. J. Agric. Environ. Ethics.

[48] Pongsilp N (2012). Phenotypic and Genotypic Diversity of Rhizobia.

[49] Ibny FY, Jaiswal SK, Mohammed M, Dakora FD (2019). Symbiotic effectiveness and ecologically adaptive traits of native rhizobial symbionts of Bambara groundnut (*Vigna subterranea* L. Verdc) in Africa and their relationship with phylogeny. Sci. Rep..

[50] Hall, T*. BioEdit version 7.0. 0*. *Distributed by the author*. https://www.mbio.ncsu.edu/BioEdit/bioedit.html (2004).

[51] Altschul SF, Gish W, Miller W, Myers EW, Lipman D (1990). Basic local alignment search tool. J. Mol. Biol..

[52] Kumar S, Stecher G, Tamura K (2016). MEGA7: Molecular evolutionary genetics analysis version 7.0 for bigger datasets. Mol. Biol. Evol..

[53] Felsenstein J (1985). Confidence limits on phylogenies: An approach using the bootstrap. Evolution.

[54] Puozaa DK, Jaiswal SK, Dakora FD (2019). Phylogeny and distribution of *Bradyrhizobium* symbionts nodulating cowpea (*Vigna unguiculata* L. Walp) and their association with the physicochemical properties of acidic African soils. Syst. Appl. Microbiol..

[55] Grönemeyer JL, Kulkarni A, Berkelmann D, Hurek T, Reinhold-Hurek B (2014). Rhizobia indigenous to the Okavango region in Sub-Saharan Africa: Diversity, adaptations, and host specificity. Appl. Environ. Microbiol..

[56] Mohammed M, Jaiswal SK, Dakora FD (2019). Insights into the phylogeny, nodule function and biogeographic distribution of microsymbionts nodulating the orphan Kersting’s groundnut [*Macrotyloma geocarpum* (Harms) Marechal & Baudet] in African soils. Appl. Environ. Microbiol..

[57] Jaiswal SK, Dakora FD (2019). Widespread distribution of highly adapted *Bradyrhizobium* species nodulating diverse legumes in Africa. Front. Microbiol..

[58] Gano-Cohen KA (2019). Interspecific conflict and the evolution of ineffective rhizobia. Ecol. Lett..

[59] Clúa J, Roda C, Zanetti ME, Blanco FA (2018). Compatibility between legumes and rhizobia for the establishment of a successful nitrogen-fixing symbiosis. Genes (Basel).

[60] Kaschuk G (2012). Photosynthetic adaptation of soybean due to varying effectiveness of N_2_ fixation by two distinct *Bradyrhizobium**japonicum* strains. Environ. Exp. Bot..

