# Supplementary material for: Functional and genetic diversity of native rhizobial isolates nodulating cowpea (Vigna unguiculata L. Walp.) in Mozambican soils
Source: Sci Rep. 2021 Jun 17;11:12747. doi: 10.1038/s41598-021-91889-7 (PMC8211668; doi:10.1038/s41598-021-91889-7)
Supplement: Supplementary file 1 — Supplementary Information. [file 41598_2021_91889_MOESM1_ESM.docx]

**Functional and genetic diversity of native rhizobial isolates nodulating cowpea (*Vigna unguiculata* L. Walp.) in Mozambican soils**

Margarida G. Simbine^1^, Mustapha Mohammed^1^**^,2,3^**, Sanjay K. Jaiswal^2^, Felix D. Dakora*^2^

^1^Department of Crop Sciences, Tshwane University of Technology, Pretoria 0001, South Africa

**^2^Department of Crop Science, University for Development Studies, P.O. Box TL1882, Tamale, Ghana**

^2^Department of Chemistry, Tshwane University of Technology, Pretoria 0001, South Africa

Table S1A Nodulation, shoot biomass, relative effectiveness and photosynthetic physiology of cowpea inoculated with rhizobia isolated from cowpea nodules sampled from AEZ 7 of the Nampula Province, Mozambique (1^st^ experiment)

| **Treatment** | **Nodule number** | **Nodule dry weight** | **Shoot dry matter** | **A** | **gs** | **Relative effectiveness** |
| --- | --- | --- | --- | --- | --- | --- |
|  | no. plant^-1^ | g plant^-1^ | g plant^-1^ | μmol (CO_2_) m^–2^ s^–1^ | mol m^–2^ s^–1^ | **%** |
| **Isolate** |  |  |  |  |  |  |
| TUTVuML6 | 67.00±10.00ghi | 0.12±0.01h-l | 3.30±0.42f-i | 23.36±0.48a-e | 0.49±0.10efg | 88.39±11.15g-j |
| TUTVuML7 | 66.00±23.00ghi | 0.16±0.01e-j | 3.17±0.69f-i | 21.06±0.54c-j | 0.52±0.00c-f | 84.82±18.58g-j |
| TUTVuML8 | 73.00±3.00f-i | 0.16±0.04f-j | 3.00±0.29f-i | 20.23±2.28f-l | 0.28±0.05i-n | 80.36±7.73g-j |
| TUTVuML12 | 58.00±14.00hij | 0.20±0.01d-h | 4.90±0.12a-d | 22.16±0.01b-g | 0.56±0.00cde | 131.25±3.09a-e |
| TUTVuML16 | 131.00±44.00cde | 0.39±0.07b | 5.03±0.38abc | 19.11±0.26i-n | 0.23±0.01l-p | 134.82±10.30a-d |
| TUTVuML18 | 72.00±2.00f-i | 0.35±0.01bc | 4.63±0.78a-e | 20.81±0.84e-k | 0.38±0.02j-g | 124.11±20.77a-f |
| TUTVuML29 | 66.00±12.00ghi | 0.17±0.01e-i | 3.70±0.12d-h | 18.25±0.62k-o | 0.22±0.00m-p | 99.11±3.09e-i |
| TUTVuML34 | 125.00±46.00c-f | 0.17±0.03e-i | 2.55±0.66hij | 24.82±0.00ab | 0.87±0.00a | 68.30±17.78ijk |
| TUTVuML35 | 64.00±20.00hij | 0.18±0.04e-h | 3.03±0.23f-i | 16.24±0.30op | 0.16±0.01nop | 81.25±6.25g-j |
| TUTVuML37 | 229.00±5.00a | 0.24±0.04de | 2.97±0.61ghi | 23.44±0.77a-e | 0.50±0.05d-g | 79.46±16.39hij |
| TUTVuML41 | 155.00±8.00bc | 0.49±0.01a | 5.55±0.55a | 16.91±0.10m-p | 0.18±0.01m-p | 148.66±14.69a |
| TUTVuML45 | 53.00±1.00hij | 0.07±0.02l | 1.55±0.19jk | 21.44±2.59c-i | 0.35±0.06h-l | 41.43±5.20k |
| TUTVuML48 | 154.00±7.00bc | 0.22±0.02d-g | 2.90±0.32ghi | 22.04±0.71c-h | 0.61±0.04cd | 77.68±8.61hij |
| TUTVuML49 | 173.00±13.00bc | 0.28±0.01cd | 3.30±0.15f-i | 23.52±0.81a-e | 0.63±0.08bc | 88.39±4.09g-j |
| TUTVuML50 | 20.00±7.00ij | 0.15±0.02f-j | 2.97±0.60ghi | 22.54±2.40b-f | 0.39±0.11ghi | 79.46±16.10hij |
| TUTVuML54 | 87.00±21.00d-h | 0.18±0.03e-h | 2.27±0.49ij | 19.79±0.40f-l | 0.26±0.01j-o | 60.71±13.15jk |
| TUTVuML55 | 142.00±21.00bcd | 0.50±0.06a | 5.33±0.67ab | 22.17±0.91b-g | 0.45±0.01e-h | 142.86±17.92ab |
| TUTVuML57 | 50.00±5.00hij | 0.23±0.01def | 3.77±0.35c-h | 19.37±0.65h-m | 0.18±0.02m-p | 100.89±9.32d-i |
| TUTVuML62 | 62.00±6.00hij | 0.23±0.02def | 4.17±0.23b-g | 22.35±0.61b-f | 0.38±0.06g-j | 111.61±6.25b-h |
| TUTVuML65 | 73.00±13.00f-i | 0.20±0.03d-h | 3.63±0.70d-h | 15.37±0.05p | 0.15±0.02op | 97.32±18.64e-i |
| TUTVuML66 | 196.00±23.00ab | 0.34±0.06bc | 4.67±0.93a-e | 11.99±0.15q | 0.12±0.00pq | 125.00±25.00a-f |
| TUTVuML74 | 83.00±35.00e-h | 0.32±0.04bc | 5.33±0.43ab | 18.60±0.21j-o | 0.16±0.01nop | 142.86±11.40ab |
| TUTVuML75 | 125.00±12.00c-f | 0.49±0.04a | 5.28±0.22ab | 17.96±0.01l-p | 0.19±0.00m-p | 141.29±5.80abc |

Table S1A Continued

| **Treatment** | **Nodule number** | **Nodule dry weight** | **Shoot dry matter** | **A** | **gs** | **Relative effectiveness** |
| --- | --- | --- | --- | --- | --- | --- |
|  | no. plant^-1^ | g plant^-1^ | g plant^-1^ | μmol (CO_2_) m^–2^ s^–1^ | mol m^–2^ s^–1^ | **%** |
| **Isolate** |  |  |  |  |  |  |
| TUTVuML76 | 137.00±27.00cde | 0.16±0.02e-j | 2.97±0.32ghi | 19.59±1.87g-m | 0.46±0.09e-h | 79.46±8.52hij |
| TUTVuMU1 | 67.00±5.00ghi | 0.16±0.03e-i | 3.97±0.20c-g | 21.70±0.39c-i | 0.36±0.02h-k | 106.25±5.43c-h |
| TUTVuMU5 | 47.00±13.00hij | 0.15±0.02f-k | 3.77±0.09c-h | 23.68±1.20abc | 0.29±0.03i-m | 100.89±2.36d-i |
| TUTVuMU6 | 48.00±2.00hij | 0.14±0.02g-l | 3.40±0.96e-i | 22.47±0.26b-f | 0.54±0.06c-f | 91.07±25.74f-j |
| TUTVuMU7 | 87.00±22.00d-h | 0.21±0.02d-g | 3.53±0.55e-i | 21.65±0.37c-i | 0.50±0.05d-g | 94.64±14.70f-j |
| TUTVuMU10 | 61.00±7.00hij | 0.14±0.02g-l | 4.30±0.64a-f | 25.46±0.42a | 0.54±0.04c-f | 115.18±17.22a-g |
| TUTVuMU12 | 28.00±5.00ij | 0.09±0.02i-l | 1.45±0.13jk | 21.67±0.11c-i | 0.45±0.00e-h | 38.80±3.39k |
| TUTVuNM7 | 120.00±46.00c-g | 0.23±0.01def | 3.20±0.06f-i | 20.92±0.95d-k | 0.53±0.06c-f | 85.71±1.55g-j |
| TUTVuNM8 | 9.00±3.00j | 0.08±0.03jkl | 1.40±0.40jk | 16.45±2.23nop | 0.49±0.05d-g | 37.50±0.98k |
| TUTVuNM10 | 35.00±0.00hij | 0.07±0.01kl | 1.37±0.07jk | 23.64±0.01a-d | 0.74±0.00b | 36.61±1.79k |
| ***B.* strain CB756** | 130.00±6.00cde | 0.19±0.01e-h | 2.94±0.07 ghi | 20.58±0.32f-l | 0.42±0.02fgh | 88.39±3.09g-j |
| **5 mM KNO_3_** | - | - | 3.51±0.11e-i | 16.10±1.03op | 0.25±0.01k-o | 100.00±11.61d-i |
| **Uninoculated** | - | - | 0.43±0.01k | 0.59±0.01r | 0.02±0.00q | - |
| ***F* statistics** | 7.16*** | 15.06*** | 7.35*** | 20.44*** | 18.46*** | 5.90*** |

Values (Means ± SE) with dissimilar letters in a column are significant at ***p≤0.001

Table S1B Nodulation, shoot biomass and photosynthetic physiology of cowpea inoculated with rhizobia isolated from cowpea nodules sampled from AEZ 7 of the Nampula Province, Mozambique (2^nd^ experiment)

| **Treatment** | **Nodule number** | **Nodule dry weight** | **Shoot dry matter** | **A** | **Gs** | **Relative effectiveness** |
| --- | --- | --- | --- | --- | --- | --- |
|  | no. plant^-1^ | g plant^-1^ | g plant^-1^ | μmol (CO_2_) m^–2^ s^–1^ | mol m^–2^ s^–1^ | **%** |
| **Isolate** |  |  |  |  |  |  |
| TUTVuML3 | 58.00±15.00i-o | 0.20±0.05h-m | 5.40±0.06abc | 18.06±0.35l-o | 0.22±0.02o-t | 144.64±1.55abc |
| TUTVuML4 | 49.00±17.00j-o | 0.13±0.08k-n | 3.90±0.80e-l | 21.32±1.14d-j | 0.44±0.08e-j | 104.55±21.38e-m |
| TUTVuML5 | 52.00±12.00j-o | 0.16±0.02i-n | 2.43±0.33n-r | 20.54±0.28g-l | 0.43±0.00e-j | 65.18±8.79o-s |
| TUTVuML9 | 43.00±13.00j-o | 0.12±0.03lmn | 1.40±0.06rs | 22.73±0.39b-g | 0.74±0.01a | 37.50±1.55s |
| TUTVuML11 | 193.00±43.00ab | 0.37±0.02b-e | 5.73±0.20a | 17.23±0.76nop | 0.17±0.01st | 153.57±5.43a |
| TUTVuML14 | 105.00±18.00e-i | 0.14±0.01j-n | 2.67±0.32m-p | 20.84±0.89f-j | 0.53±0.01cde | 71.43±8.52n-r |
| TUTVuML19 | 90.00±4.00f-k | 0.32±0.02c-g | 5.27±0.55a-d | 19.39±0.71i-n | 0.19±0.01q-t | 141.07±14.70a-d |
| TUTVuML21 | 116.00±21.00d-h | 0.19±0.04h-m | 3.33±0.27h-o | 23.37±0.91a-e | 0.50±0.06c-g | 89.29±7.31h-p |
| TUTVuML23 | 37.00±7.00l-o | 0.13±0.02k-n | 2.64±0.21m-q | 21.42±0.00d-i | 0.19±0.00q-t | 70.80±5.59n-r |
| TUTVuML24 | 75.00±27.00g-o | 0.24±0.16g-k | 1.46±0.09qrs | 17.21±1.97nop | 0.35±0.05i-n | 39.11±2.53rs |
| TUTVuML25 | 122.00±12.00d-g | 0.31±0.07d-h | 5.33±0.82a-d | 20.02±0.13h-m | 0.16±0.01st | 142.86±22.00a-d |
| TUTVuML26 | 62.00±6.00i-o | 0.21±0.02g-l | 3.93±0.69e-k | 15.24±0.76pq | 0.23±0.07o-t | 105.36±18.58e-l |
| TUTVuML27 | 147.00±37.00b-e | 0.48±0.08ab | 5.77±0.33a | 20.82±1.38f-j | 0.40±0.06g-k | 154.46±8.93a |
| TUTVuML28 | 175.00±18.00abc | 0.19±0.01h-m | 4.77±0.15a-f | 22.22±0.85b-h | 0.39±0.03g-l | 127.68±3.89a-f |
| TUTVuML30 | 93.00±9.00f-j | 0.21±0.04g-l | 4.70±0.51a-g | 22.41±0.77b-h | 0.33±0.02j-o | 125.89±13.75a-g |
| TUTVuML31 | 116.00±9.00d-h | 0.25±0.01f-j | 4.70±0.10a-g | 15.26±1.23pq | 0.14±0.02t | 125.89±2.68a-g |
| TUTVuML32 | 67.00±6.00h-o | 0.18±0.03i-m | 4.37±0.48b-i | 20.62±0.31g-k | 0.36±0.07h-m | 116.96±12.97b-i |
| TUTVuML33 | 215.00±10.00a | 0.22±0.01g-l | 4.17±0.43d-k | 23.73±0.81a-d | 0.66±0.06ab | 111.61±11.40d-k |
| TUTVuML36 | 108.00±12.00d-i | 0.19±0.01i-m | 3.73±0.07f-m | 21.59±0.18d-i | 0.51±0.01c-f | 100.00±1.79f-n |
| TUTVuML38 | 41.00±6.00k-o | 0.15±0.01j-n | 3.53±0.28g-n | 21.13±0.13e-j | 0.47±0.00d-h | 94.64±7.63g-o |
| TUTVuML39 | 26.00±0.00no | 0.16±0.00i-n | 2.20±0.23o-r | 20.09±0.73h-m | 0.30±0.01k-q | 58.93±6.19p-s |
| TUTVuML42 | 183.00±33.00abc | 0.22±0.01g-l | 3.20±0.29i-o | 22.70±0.27b-g | 0.59±0.04bc | 85.71±7.73i-p |
| TUTVuML43 | 152.00±17.00b-e | 0.24±0.01g-k | 3.63±0.33f-m | 21.95±0.25c-h | 0.53±0.05cde | 97.32±8.79f-o |
| TUTVuML44 | 24.00±0.00o | 0.05±0.02n | 1.47±0.12qrs | 25.33±1.28a | 0.40±0.05f-k | 39.29±3.22rs |
| TUTVuML46 | 71.00±16.00g-o | 0.31±0.05d-h | 3.73±0.54f-m | 17.06±1.06nop | 0.18±0.03rst | 100.00±14.37f-n |

Table S1B Continued

| **Treatment** | **Nodule number** | **Nodule dry weight** | **Shoot dry matter** | **A** | **Gs** | **Relative effectiveness** |
| --- | --- | --- | --- | --- | --- | --- |
|  | no. plant^-1^ | g plant^-1^ | g plant^-1^ | μmol (CO_2_) m^–2^ s^–1^ | mol m^–2^ s^–1^ | **%** |
| **Isolate** |  |  |  |  |  |  |
| TUTVuML47 | 174.00±53.00abc | 0.36±0.04b-f | 5.17±0.59a-d | 18.25±2.49k-o | 0.20±0.04p-t | 138.39±15.87a-d |
| TUTVuML51 | 27.00±6.00no | 0.09±0.03mn | 1.46±0.26qrs | 15.98±1.27opq | 0.33±0.05j-o | 38.99±6.96rs |
| TUTVuML53 | 48.00±14.00j-o | 0.13±0.01k-n | 3.27±0.26i-o | 18.87±0.40j-n | 0.29±0.04k-r | 87.50±6.97h-p |
| TUTVuML56 | 88.00±19.00g-l | 0.27±0.03e-i | 4.47±0.41b-h | 17.47±0.59nop | 0.26±0.05m-s | 119.64±10.97b-h |
| TUTVuML58 | 58.00±9.00i-o | 0.16±0.01i-n | 3.60±0.26f-n | 20.83±0.15f-j | 0.36±0.03h-m | 96.43±7.09f-o |
| TUTVuML60 | 61.00±3.00i-o | 0.19±0.04h-m | 4.27±0.41c-j | 24.64±1.44ab | 0.56±0.03bcd | 114.29±10.97c-j |
| TUTVuML61 | 43.00±5.00j-o | 0.16±0.04i-n | 3.70±0.67f-m | 21.76±0.21c-i | 0.30±0.04k-q | 99.11±17.83f-n |
| TUTVuML63 | 122.00±8.00d-g | 0.50±0.02a | 5.75±0.26a | 23.36±0.09a-e | 0.31±0.01k-p | 154.02±6.96a |
| TUTVuML64 | 90.00±25.00f-k | 0.24±0.05g-k | 2.70±0.74m-p | 18.84±1.66j-n | 0.28±0.02l-s | 72.32±19.74m-q |
| TUTVuML68 | 49.00±8.00j-o | 0.13±0.04k-n | 3.00±0.64k-p | 17.22±0.27nop | 0.17±0.01st | 80.36±17.01k-q |
| TUTVuML69 | 81.00±27.00g-m | 0.11±0.04lmn | 3.17±0.03j-o | 17.73±0.09m-p | 0.17±0.00st | 84.82±0.89i-p |
| TUTVuML70 | 156.00±5.00bcd | 0.19±0.00h-m | 3.50±0.17h-n | 22.77±0.61b-g | 0.52±0.06cde | 93.75±4.64g-o |
| TUTVuML71 | 40.00±4.00k-o | 0.14±0.00j-n | 2.73±0.33l-p | 22.82±0.17b-g | 0.46±0.07d-i | 73.21±8.93l-q |
| TUTVuML73 | 140.00±15.00c-f | 0.40±0.11a-d | 3.07±0.58k-p | 22.94±1.02a-g | 0.30±0.03k-q | 82.14±15.49j-q |
| TUTVuMU2 | 67.00±34.00h-o | 0.14±0.05j-n | 1.40±0.40rs | 20.68±0.92f-k | 0.30±0.02k-q | 37.50±10.71s |
| TUTVuMU3 | 45.00±28.00j-o | 0.20±0.02h-m | 3.13±0.33j-o | 14.25±0.73q | 0.18±0.03rst | 83.93±8.93j-p |
| TUTVuMU4 | 75.00±11.00g-o | 0.40±0.06a-d | 5.00±0.85a-e | 18.24±0.75k-o | 0.24±0.04n-t | 133.93±22.78a-e |
| TUTVuMU9 | 51.00±22.00j-o | 0.16±0.03i-n | 3.73±0.84f-m | 24.07±1.46abc | 0.43±0.11e-j | 100.00±22.53f-n |
| TUTVuMU13 | 122.00±11.00d-g | 0.43±0.03abc | 5.53±0.27ab | 13.68±0.00q | 0.14±0.00t | 148.21±7.31ab |
| TUTVuNM6 | 36.00±0.00mno | 0.13±0.00k-n | 1.90±0.35pqr | 22.87±0.05a-g | 0.48±0.00c-g | 50.89±9.28qrs |
| TUTVuNM9 | 76.00±3.00g-n | 0.18±0.01i-m | 3.63±0.41f-m | 23.16±1.29a-f | 0.46±0.08d-i | 97.32±10.86f-o |
| ***B.* strain CB756** | 119.00±8.00d-g | 0.21±0.02g-m | 3.10±0.09j-o | 22.50±0.01b-h | 0.56±0.05bcd | 88.39±3.09h-p |
| **5 mM KNO_3_** | - | - | 3.68±0.06f-m | 16.92±0.97nop | 0.23±0.03o-t | 100.00±11.61f-n |
| **Uninoculated** | - | - | 0.45±0.04s | 0.63±0.01r | 0.02±0.00u | - |
| ***F* statistics** | 7.10*** | 5.71*** | 9.46*** | 20.51*** | 14.62*** | 9.21*** |

Values (Means ± SE) with dissimilar letters in a column are significant at ***p≤0.001

Table S2 Nodulation, shoot biomass and photosynthetic physiology of cowpea inoculated with rhizobia isolated from cowpea nodules sampled from AEZ 8 of the Nampula Province, Mozambique

| **Treatment** | **Nodule number** | **Nodule dry weight** | **Shoot dry matter** | **A** | **gs** | **Relative effectiveness** |
| --- | --- | --- | --- | --- | --- | --- |
|  | no. plant^-1^ | g plant^-1^ | g plant^-1^ | μmol (CO_2_) m^–2^ s^–1^ | mol m^–2^ s^–1^ | **%** |
| **Isolate** |  |  |  |  |  |  |
| TUTVuAG1 | 168.00±3.00d-n | 0.43±0.02a-e | 4.77±0.32a-g | 21.10±0.25g-p | 0.41±0.02i-q | 127.69±8.52a-g |
| TUTVuAG2 | 206.00±16.00b-j | 0.39±0.02a-k | 4.60±0.26c-k | 21.81±1.22c-n | 0.49±0.02e-m | 123.23±7.09b-k |
| TUTVuAG3 | 158.00±13.00g-n | 0.30±0.02g-p | 3.63±0.55k-q | 21.82±0.76c-n | 0.46±0.07e-o | 97.33±14.70k-q |
| TUTVuAG4 | 225.00±34.00a-i | 0.35±0.05c-n | 5.13±0.37a-e | 22.04±0.70c-n | 0.50±0.05d-l | 137.51±9.94a-e |
| TUTVuAG6 | 223.00±16.00a-i | 0.30±0.05f-p | 4.43±0.52c-l | 21.60±0.56d-o | 0.41±0.04i-q | 118.76±14.03c-l |
| TUTVuAG7 | 177.00±28.00d-n | 0.32±0.07d-o | 3.73±0.17i-q | 21.82±1.67c-n | 0.39±0.02l-q | 100.01±4.46i-q |
| TUTVuAG8 | 56.00±11.00p | 0.28±0.07i-p | 4.50±0.35c-l | 21.22±0.52f-o | 0.48±0.00e-n | 120.55±9.28c-l |
| TUTVuAG10 | 100.00±8.00m-q | 0.25±0.01n-r | 3.37±0.19m-r | 23.23±0.04a-e | 0.44±0.03g-q | 90.19±4.97m-r |
| TUTVuAG11 | 286.00±32.00abc | 0.34±0.05c-n | 5.57±0.52ab | 21.78±0.99c-n | 0.43±0.08h-q | 149.12±13.95ab |
| TUTVuAG12 | 150.00±22.00i-n | 0.35±0.06b-n | 4.17±0.09e-m | 22.91±0.35a-h | 0.62±0.04abc | 111.62±2.36e-m |
| TUTVuAG14 | 246.00±37.00a-g | 0.47±0.10ab | 4.70±0.30b-i | 21.13±0.06g-p | 0.39±0.02l-q | 125.90±8.04b-i |
| TUTVuAG15 | 200.00±31.00c-k | 0.31±0.02e-o | 4.67±0.44b-j | 21.63±0.66d-o | 0.54±0.02a-h | 125.01±11.71b-j |
| TUTVuAG16 | 204.00±9.00b-k | 0.35±0.03b-n | 4.57±0.44c-k | 20.75±0.75j-r | 0.34±0.02p-q | 122.33±11.81c-k |
| TUTVuAG17 | 168.00±9.00d-n | 0.41±0.03a-g | 5.37±0.15abc | 21.36±0.54e-o | 0.38±0.05m-q | 143.76±3.89abc |
| TUTVuAG19 | 219.00±37.00b-i | 0.39±0.03a-l | 5.23±0.62abc | 22.93±0.76a-g | 0.36±0.03o-q | 140.19±16.68abc |
| TUTVuAG20 | 184.00±12.00d-m | 0.27±0.02k-q | 3.77±0.20h-q | 20.45±0.80l-r | 0.45±0.01f-p | 100.90±5.43h-q |
| TUTVuAG21 | 141.00±22.00i-p | 0.33±0.02d-o | 3.40±0.60m-r | 20.97±1.32i-r | 0.40±0.06j-q | 91.08±16.07m-r |
| TUTVuAG24 | 61.00±17.00op | 0.17±0.01p-s | 2.93±0.55p-s | 20.14±0.79n-s | 0.34±0.05p-q | 78.58±14.62p-t |
| TUTVuIM1 | 152.00±4.00h-n | 0.15±0.01qrs | 4.17±0.28e-m | 20.18±1.52m-s | 0.44±0.04g-q | 111.62±7.63e-m |
| TUTVuIM2 | 307.00±14.00a | 0.34±0.07c-o | 5.20±0.45abc | 21.92±0.71c-n | 0.53±0.05b-h | 139.30±12.08abc |
| TUTVuIM3 | 54.00±14.00p | 0.14±0.01rs | 1.67±0.28t | 20.78±0.35j-r | 0.43±0.04h-q | 44.65±7.63u |
| TUTVuIM6 | 147.00±24.00i-o | 0.36±0.08a-n | 3.97±0.24f-n | 23.44±0.13a-d | 0.55±0.05a-g | 106.26±6.44f-n |
| TUTVuIM7 | 109.00±18.00l-p | 0.35±0.01b-n | 3.70±0.55j-q | 22.79±0.37a-i | 0.54±0.04a-h | 99.12±14.75j-q |

Table S2 Continued

| **Treatment** | **Nodule number** | **Nodule dry weight** | **Shoot dry matter** | **A** | **gs** | **Relative effectiveness** |
| --- | --- | --- | --- | --- | --- | --- |
|  | no. plant^-1^ | g plant^-1^ | g plant^-1^ | μmol (CO_2_) m^–2^ s^–1^ | mol m^–2^ s^–1^ | **%** |
| **Isolate** |  |  |  |  |  |  |
| TUTVuIM8 | 250.00±12.00a-e | 0.29±0.07g-p | 4.57±0.38c-k | 22.82±0.43a-i | 0.44±0.00g-q | 122.33±10.30c-k |
| TUTVuIM9 | 249.00±9.00a-f | 0.28±0.04j-q | 2.97±0.54o-s | 21.30±0.03e-o | 0.43±0.01h-q | 79.47±14.37o-t |
| TUTVuIM12 | 256.00±32.00a-d | 0.29±0.09g-p | 4.20±0.35d-m | 22.12±0.22c-m | 0.53±0.06b-h | 112.51±9.28d-m |
| TUTVuIM13 | 184.00±9.00d-m | 0.26±0.08l-r | 3.90±0.42f-p | 22.54±0.66c-k | 0.52±0.07b-i | 104.47±11.15f-p |
| TUTVuIM14 | 226.00±20.00a-i | 0.38±0.04a-m | 2.90±0.47qrs | 19.23±0.60p-s | 0.45±0.38f-p | 77.69±12.66q-t |
| TUTVuIM15 | 171.00±19.00d-n | 0.40±0.04a-k | 2.27±0.15st | 22.05±0.28c-n | 0.41±0.01i-q | 60.72±3.89stu |
| TUTVuIM16 | 183.00±15.00d-m | 0.34±0.04c-o | 4.17±0.48e-m | 22.29±0.39c-k | 0.52±v0.01c-j | 111.62±12.88e-m |
| TUTVuIM17 | 288.00±40.00ab | 0.42±0.03a-f | 4.87±0.46a-f | 21.00±1.31g-q | 0.47±0.13e-o | 130.37±12.41a-f |
| TUTVuIM18 | 226.00±39.00a-i | 0.39±0.01a-k | 5.00±0.25a-e | 22.08±0.51c-n | 0.51±0.06c-k | 133.94±6.74a-e |
| TUTVuIM19 | 211.00±35.00b-j | 0.45±0.02a-d | 3.40±0.10m-r | 21.21±0.47f-o | 0.44±0.04g-q | 91.08±2.68m-r |
| TUTVuIM20 | 117.00±18.00k-p | 0.34±0.04c-n | 3.40±0.10m-r | 19.71±0.64o-s | 0.48±0.05e-o | 91.08±2.68m-r |
| TUTVuIM22 | 239.00±12.00a-h | 0.40±0.01a-j | 5.13±0.19a-e | 23.08±0.66a-f | 0.56±0.05a-f | 137.51±4.97a-e |
| TUTVuIM23 | 161.00±27.00g-n | 0.21±0.03o-r | 3.53±0.28l-q | 19.03±1.14rs | 0.46±0.02e-o | 94.65±7.63l-q |
| TUTVuIM24 | 207.00±25.00b-j | 0.30±0.05f-p | 4.80±0.10a-g | 21.25±0.55f-o | 0.45±0.04f-p | 128.58±2.68a-g |
| TUTVuIM25 | 161.00±21.00f-n | 0.46±0.03abc | 4.60±0.50c-k | 18.41±0.08s | 0.33±0.03q | 123.23±13.48b-k |
| TUTVuMS1 | 288.00±41.00ab | 0.37±0.03a-m | 3.93±0.33f-o | 21.92±1.03c-n | 0.56±0.56a-f | 105.37±8.79f-o |
| TUTVuMS2 | 146.00±11.00i-o | 0.30±0.03f-p | 5.30±0.40abc | 21.77±0.75c-n | 0.46±0.06e-o | 141.98±10.83abc |
| TUTVuMS3 | 184.00±28.00d-m | 0.35±0.04b-n | 2.50±0.15rst | 22.11±0.13c-m | 0.48±0.02e-o | 66.97±4.09r-u |
| TUTVuMS6 | 201.00±17.00b-k | 0.32±0.07d-o | 4.73±0.22a-h | 23.63±0.38abc | 0.53±0.03b-h | 126.80±5.86a-h |
| TUTVuMS7 | 200.00±28.00b-k | 0.41±0.03a-i | 3.33±0.46m-r | 22.36±1.10c-k | 0.57±0.04a-e | 89.29±12.41m-r |
| TUTVuMS8 | 182.00±28.00d-m | 0.33±0.05d-o | 3.87±0.18g-q | 22.05±0.89c-n | 0.48±0.10e-o | 103.58±4.72g-q |
| TUTVuMS9 | 168.00±24.00d-n | 0.35±0.02c-n | 4.63±0.12b-j | 21.01±0.57g-q | 0.39±0.05l-q | 124.12±3.22b-j |
| TUTVuMS10 | 90.00±3.00nop | 0.08±0.01s | 1.75±0.03t | 22.47±0.28c-k | 0.54±0.00b-h | 46.88±0.77u |
| TUTVuMS11 | 159.00±24.00g-n | 0.35±0.06b-n | 2.13±0.15st | 23.67±0.63abc | 0.66±0.04a | 57.15±3.89tu |

Table S2 Continued

| **Treatment** | **Nodule number** | **Nodule dry weight** | **Shoot dry matter** | **A** | **gs** | **Relative effectiveness** |
| --- | --- | --- | --- | --- | --- | --- |
|  | no. plant^-1^ | g plant^-1^ | g plant^-1^ | μmol (CO_2_) m^–2^ s^–1^ | mol m^–2^ s^–1^ | % |
| **Isolate** |  |  |  |  |  |  |
| TUTVuMS12 | 139.00±27.00i-p | 0.35±0.06b-n | 2.07±0.09st | 24.58±0.05a | 0.55±0.06a-g | 55.36±2.36tu |
| TUTVuMS13 | 204.00±15.00b-k | 0.33±0.03c-o | 1.67±0.07t | 22.60±0.97b-j | 0.64±0.04ab | 44.65±1.79u |
| TUTVuMS14 | 198.00±34.00c-k | 0.33±0.10c-o | 3.00±0.40n-s | 23.41±0.20a-d | 0.40±0.01k-q | 80.36±10.83n-t |
| TUTVuMS17 | 218.00±44.00b-i | 0.41±0.06a-h | 5.10±0.36a-e | 22.41±0.44c-k | 0.48±0.01e-n | 136.62±9.66a-e |
| TUTVuMS18 | 217.00±26.00b-i | 0.48±0.01a | 3.24±0.12m-r | 24.50±1.04ab | 0.48±0.02e-o | 86.79±3.27m-s |
| TUTVuMS19 | 189.00±31.00d-l | 0.31±0.01e-o | 4.67±0.19b-j | 22.12±0.44c-m | 0.52±0.05c-j | 125.01±4.97b-j |
| TUTVuMS20 | 156.00±9.00h-n | 0.29±0.02g-p | 4.57±0.56c-k | 20.77±0.73h-r | 0.48±0.02e-n | 122.33±15.02c-k |
| TUTVuMS22 | 165.00±17.00e-n | 0.28±0.05h-p | 5.17±0.32a-d | 22.03±0.46c-n | 0.39±0.04l-q | 138.41±8.52a-d |
| TUTVuMS23 | 220.00±44.00a-i | 0.26±0.01m-r | 4.00±0.35f-m | 21.64±0.18d-o | 0.41±0.04i-q | 107.15±9.41f-m |
| TUTVuMS24 | 158.00±36.00h-n | 0.27±0.04j-q | 5.57±0.03ab | 20.62±0.40k-r | 0.37±0.03n-q | 149.12±0.89ab |
| TUTVuMS25 | 171.00±2.00d-n | 0.35±0.02b-n | 5.70±0.21a | 20.97±0.37i-r | 0.38±0.01m-q | 152.69±5.58a |
| ***B.* strain CB756** | 123.00±11.00j-p | 0.22±0.01o-r | 3.30±0.12m-r | 23.22±0.42a-e | 0.62±0.01a-d | 88.40±3.09m-r |
| **5 mM KNO_3_** | - | - | 3.73±0.04i-q | 19.08±0.36qrs | 0.40±0.01k-q | 100.00±11.61i-q |
| **Uninoculated** | - | - | 0.48±0.43u | 0.88±0.03t | 0.05±0.00r | - |
| ***F statistics*** | 3.21*** | 3.14*** | 10.55*** | 18.09*** | 4.82*** | 10.55*** |

Values (Means ± SE) with dissimilar letters in a column are significant at ***p≤0.001

Table S3 Significant correlation coefficients between some important symbiotic effectiveness and photosynthetic physiology traits in cowpea inoculated with effective isolates from AEZ 7 and AEZ 8

| **Origen of Isolates** | **Traits** | **r-value** | **p-value** |
| --- | --- | --- | --- |
| **AEZ 7** | **1^st^ experiment** | | |
|  | Nodule number per plant and nodule dry weight per plant | 0.6328 | *** |
|  | Nodule number per plant and shoot dry matter per plant | 0.4018 | *** |
|  | Nodule dry weight per plant and shoot dry matter per plant | 0.6349 | *** |
|  |  | | |
|  | **2^nd^ experiment** | | |
|  | Nodule number per plant and nodule dry weight per plant | 0.6431 | *** |
|  | Nodule number per plant and shoot dry matter per plant | 0.4723 | *** |
|  | Nodule dry weight per plant and shoot dry matter per plant | 0.6071 | *** |
|  |  |  |  |
| **AEZ 8** | Nodule number per plant and nodule dry weight per plant | 0.5801 | *** |
|  | Nodule number per plant and shoot dry matter per plant | 0.3543 | *** |
|  | Nodule dry weight per plant and shoot dry matter per plant | 0.2996 | *** |

Correlations are significant at **p≤0.01 and ***p≤0.001
